# Building Molecules by a Self‐Replicator That Catalyzes Acyl Hydrazone Formation

**DOI:** 10.1002/anie.202506986

**Published:** 2026-01-24

**Authors:** Kayleigh S. van Esterik, Tommaso Marchetti, Sijbren Otto

**Affiliations:** ^1^ Centre for Systems Chemistry Stratingh Institute for Chemistry University of Groningen Nijenborgh 3, 9747 AG Groningen the Netherlands

**Keywords:** Acyl hydrazone formation, De‐novo life, Self‐replication, Supramolecular catalysis, Systems chemistry

## Abstract

Catalysis of bond‐forming reactions is key to the development of life‐like chemical systems as it allows to build up new material, increasing molecular complexity and diversity. Integrating catalysis with other characteristic properties of life, like self‐replication, represents an important advance in the transition from chemistry to life. We have previously shown that catalysis can emerge in synthetic self‐replicators that form through supramolecular assembly. However, the organocatalyzed reactions were solely bond‐breaking so far. We now report the successful expansion of the catalytic promiscuity of these systems to bond‐forming reactions. We show that a self‐replicator efficiently catalyzes acyl hydrazone formation between different hydrazides and aldehydes. This marks an important step towards the further development of evolvable systems that combine metabolic activity with self‐replication.

## Graphical abstract

A self‐replicator efficiently catalyzes the formation of acyl hydrazone bonds between a set of different substrates. In addition to previously reported (bond‐breaking) catalytic activity, this renders the self‐replicator highly promiscuous, which is a good starting point for Darwinian experiments en route to de‐novo life.

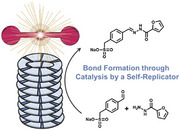

Understanding the transition from chemistry to life is one of the major questions in science.^[^ ^1^, ^2^, ^3^, ^4^ ^]^ Experimental approaches to this question involve devising synthetic chemical systems that implement key characteristics of life such as self‐replication, metabolism or compartmentalization and then continue towards their pairwise integration and beyond.^[^ ^5^, ^6^, ^7^, ^8^, ^9^, ^10^, ^11^ ^]^ Systems that aim for the integration of metabolism feature catalysis as a key trait, as metabolism relies on the acceleration of reactions. For example, research that starts to address the pairwise combination of metabolism with compartmentalization through catalysis has utilized autocatalytic networks,^[^ ^12^, ^13^ ^]^ enzymatic activity,^[^ ^14^, ^15^, ^16^, ^17^, ^18^, ^19^, ^20^, ^21^, ^22^ ^]^ or the internal microenvironment of compartments.^[^ ^23^, ^24^, ^25^, ^26^, ^27^, ^28^, ^29^, ^30^ ^]^

Chemical self‐replicators autonomously catalyze the formation of copies of themselves while transferring information to the next generation, typically through templation.^[^ ^8^, ^31^, ^32^, ^33^ ^]^ Reports that start to explore their combination with metabolism feature replicators that are able to catalyze chemical reactions other than their own replication.^[^ ^34^, ^35^, ^36^, ^37^ ^]^ Yet, these examples almost exclusively concern systems where molecules are being broken down, whereas it would be more desirable to build up material and transform simple molecules into more complex ones. This would allow for further complexification and diversification of a chemical system. These are important features in the transition of chemistry into life that cannot be achieved by catalysis of bond‐breaking reactions alone. Still, reports of self‐replicators that catalyze exogenous bond‐forming reactions are rare. A pioneering example by Rebek and coworkers combined a synthetic self‐replicator with catalysis of a bond‐forming reaction, but required conditions for the latter that were not compatible with replication (different solvent system).^[^ ^34^ ^]^

We previously reported self‐replicators^[^ ^38^, ^39^ ^]^ capable of catalyzing bond‐breaking reactions (FMOC deprotection, retro‐aldol catalysis)^[^ ^35^ ^]^ and able to recruit a cofactor to accelerate disulfide formation.^[^ ^36^ ^]^ These studies featured replicators made from building block **1** consisting of a dithiol benzene core with a pentapeptide (GLKFK) attached to it. Upon dissolution in an aqueous buffer and oxidation by air, macrocycles of various sizes form by disulfide formation. These macrocycles, dominated by trimers (**1_3_**) and tetramers (**1_4_**), continuously interconvert through disulfide exchange. Over time, hexamers (**1_6_**) assemble into fibers through a nucleation‐growth mechanism, facilitated by the presence of reservoirs of macrocycles on the surfaces of formed fibers (Figure 1a, b).^[^ ^40^ ^]^ Continuous disulfide exchange in the macrocycle pool replenishes hexamers available for self‐assembly into fibers, though we cannot exclude a catalytic role of fiber ends in hexamer formation. The self‐assembly of a specific macrocycle thus drives its self‐replication. The replication process becomes exponential when mechanical agitation is applied, as fiber breakage increases the number of growing ends.^[^ ^41^ ^]^

**Figure 1:**
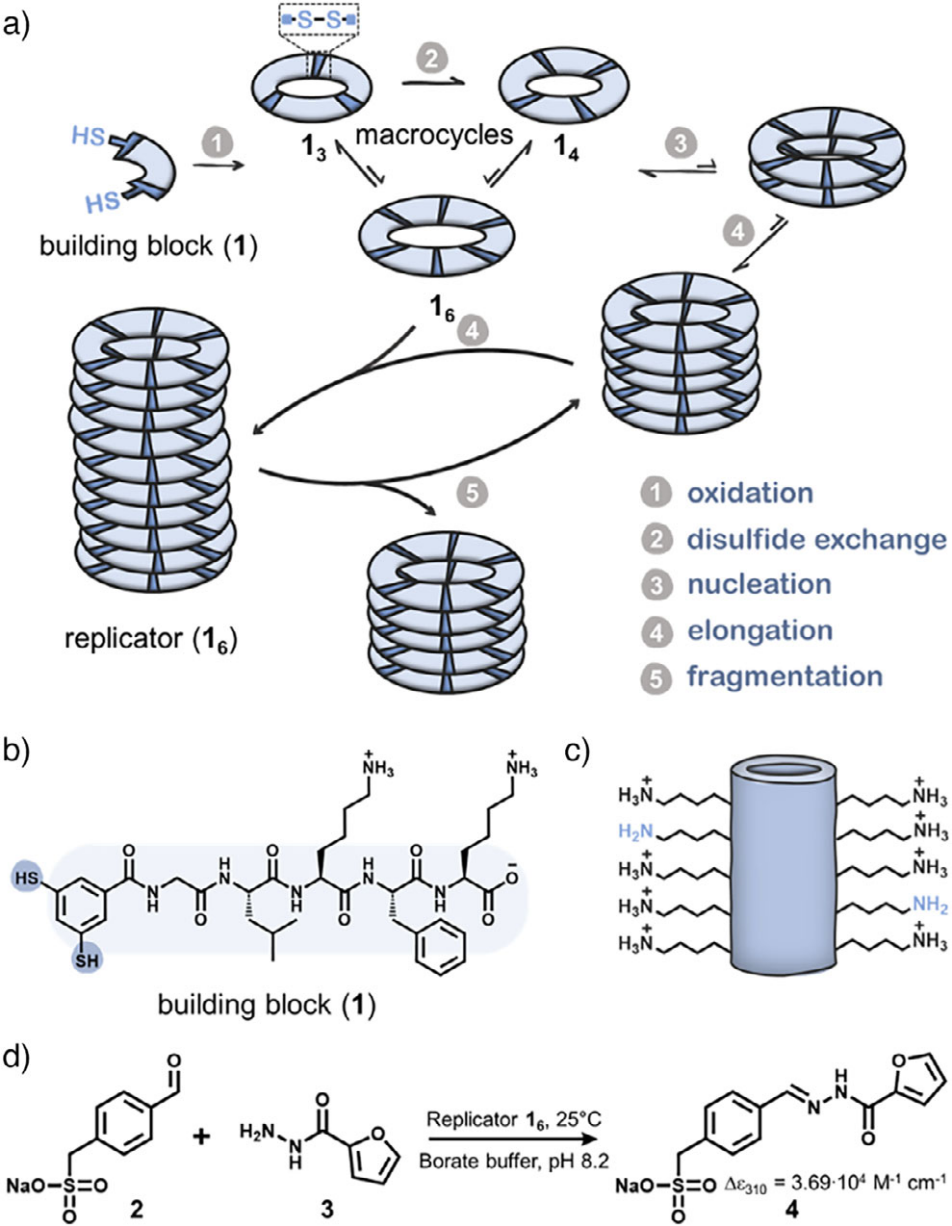
a) Schematic representation of the self‐replication mechanism, b) structural formula of dithiol building block **1**, c) the high density of lysine side chains in fibers formed from **1**_6_ perturbs the *pK_a_* of the amine groups such that some are unprotonated and catalytically active, d) acyl hydrazone formation catalyzed by self‐replicator **1_6_**.

The structural organization that takes place when replicators self‐assemble is crucial for catalysis. Fiber formation brings the charged ε‐amines of the lysines sidechains of the replicator building block into close proximity, which perturbs their *pK_a_* to reduce unfavorable electrostatic interactions (Figure 1c). This effect increases the number of non‐protonated amines, which can then take part in nucleophilic catalysis, for instance through iminium ion formation with an aldehyde or ketone.^[^ ^35^, ^42^, ^43^, ^44^ ^]^ Inspired by previous work on organocatalytic polymers^[^ ^45^ ^]^ and catalysis of bond‐formation by supramolecular structures,^[^ ^46^, ^47^, ^48^, ^49^, ^50^, ^51^ ^]^ we hypothesized that it should be possible to exploit the presence of non‐protonated lysines to also catalyze acyl hydrazone formation. Though acyl hydrazone formation typically requires Brønsted acid catalysis (i.e., typically pH 4.5‐5), its formation can also be promoted at neutral pH through iminium catalysis.^[^ ^45^, ^50^, ^51^, ^52^, ^53^, ^54^, ^55^ ^]^

We now show that, in addition to previously reported catalysis of bond‐breaking reactions, our self‐replicators also catalyze acyl hydrazone formation. This further increases their catalytic promiscuity and allows for building up new material of increased structural complexity, which are important features in the further development of synthetic life‐like systems.

We specifically aimed for a well water‐soluble pair of both reaction substrates and acyl hydrazone product to ease analysis and quantification of the reaction. Therefore, we developed a spectrophotometric assay for acyl hydrazone formation using a negatively charged aldehyde **2**, which, after hydrazone formation with hydrazide **3** (chosen inspired by literature^[^ ^56^ ^]^) yields a well water‐soluble hydrazone (**4**) that is readily quantifiable with UV‐vis spectrophotometry (Figure 1d & Figure S1, Δε_310_ = 3.69·10^4^ M^−1^ cm^−1^ in borate buffer (pH 8.2)). Next to ensuring good water solubility, a negatively charged aldehyde is expected to have favorable electrostatic interactions with the positively charged replicator fibers.

Indeed, when combining substrates **2** (50 µM) and **3** (0.20 mM) with replicator **1_6_** (50 µM expressed in building block **1**) in borate buffer (50 mM, pH 8.2), we observed rapid acyl hydrazone formation (Figure 2a). Moreover, self‐replicator **1_6_** outperforms precursors **1** and **1_3/4_** in the catalysis of this reaction. While monomeric building block **1** performed similarly to the blank, some activity is observed for **1_3/4_** macrocycles. We ascribe this to some degree of activation of the ε‐amines of the lysines as these macrocycles are not molecularly dissolved, but form ill‐defined aggregates in solution.^[^ ^57^ ^]^ Formation of **4** was confirmed with negative‐mode mass spectrometry and comparison of retention time and UV spectrum to a synthesized reference compound by ultra‐performance liquid chromatography (UPLC) (Figure S6). The ability of **1_6_** to catalyze more than one turnover was confirmed by monitoring the formation of **4** in presence of a lower concentration of **1_6_** (10 µM) in otherwise identical conditions. Analysis by TEM indicated that the structural integrity of replicator fibers is not affected significantly by the hydrazone formation reaction (Figure S7).

**Figure 2:**
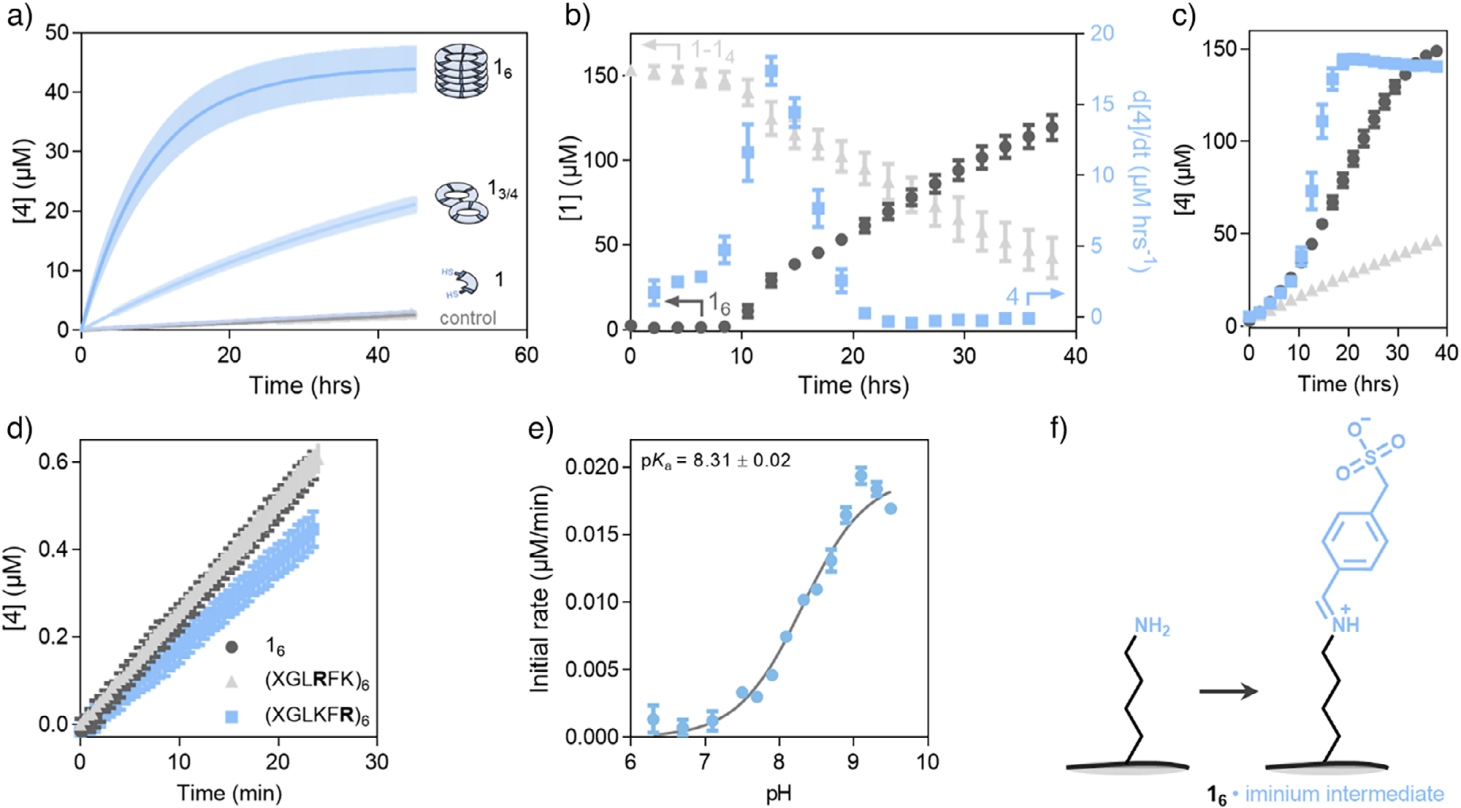
a) The formation of **4** from **2** (50 µM) and **3** (0.20 mM) is faster in presence of **1_6_** (50 µM with respect to **1**) than in presence of **1** (50 µM), a mixture of **1_3_** and **1_4_** (**1_3/4_**, 50 µM), or control without catalyst. Measurements were conducted spectrophotometrically (Δε_310_ = 3.69·10^4^ M^−1^ cm^−1^) as independent duplicates and error bars represent a single standard deviation. b) Acceleration of the rate of formation of **4** from **2** (0.20 mM) and **3** (0.80 mM) coincides with the onset of **1_6_** formation in an emergence experiment from a stirred (1200 rpm) mixture of **1** (0.20 mM). Measurements were conducted using an automated stirring device inside a UPLC^[^ ^59^ ^]^ at 40°C. Peak areas corresponding to **1_6_,** precursors **1**–**1_4_** and **4** were converted to concentration or rate of formation using a calibration curve (at 254 nm for **1** and 340 nm for **4**, Figure S1). Measurements were conducted in duplicate and error bars represent a single standard deviation. Controls in absence of **1** or without stirring are shown in Figure S8, S9. c) Product formation of **4** in the experiment of panel b, in the absence of **1** (light grey triangles), in the presence of **1** without stirring (dark grey circles) or presence of **1** with stirring (light blue squares). d) Initial formation of **4** from **2** (50 µM) and **3** (200 µM) catalyzed by hexamer replicators (25 µM) with the inner (XGL**R**FK) or the outer (XGLKF**R**) lysine replaced with arginine, measured in triplicate. Error bars represent a single standard deviation. e) A pH‐rate profile obtained for catalysis of acyl hydrazone formation from **2** (50 µM) and **3** (200 µM) by **1_6_** (25 µM), measured in triplicate. Error bars represent a single standard deviation. f) Proposed iminium ion intermediate at the lysine sidechains of **1_6_**.

We performed the spectrophotometric measurements by pre‐incubating **1_6_** with aldehyde **2** before the addition of hydrazide **3**. This procedure avoided the small non‐linear increase in product formation during the initial phase of the reaction that was observed when **1_6_** was added to a pre‐incubated solution of **2** and **3**. We did not investigate this further as the effect was very small (Figure S13). While iminium ion formation can result in a changed UV absorbance,^[^ ^58^ ^]^ incubating **1_6_** with **2** did not show an appreciable change in the UV spectrum (Figure S1e).

To confirm that acyl hydrazone catalysis is an emergent property of the replicator system, we added monomeric building block **1** (final concentration 0.20 mM) to a solution of **2** (0.20 mM) and **3** (0.80 mM) in borate buffer (pH 8.2) at 40°C under mechanical agitation (stirring at 1200 rpm) and monitored the formation of replicator **1_6_** and **4** over time by UPLC. Even though some hydrolysis of **4** seems to take place in the acidic conditions during UPLC analysis (Figure S6), the results clearly show that the onset of accelerated acyl hydrazone formation coincided with the emergence of **1_6_** (Figure 2b). As mechanical agitation is required to break replicator fibers and thus increase the number of fibers growing ends, the formation of **1_6_** is suppressed without stirring.^[^ ^41^ ^]^ The absence of stirring resulted in slower **4** formation and more replicator precursor material remained present for longer (Figure S8a, S9). Moreover, in the time frame prior to the emergence of **1_6_**, the formation of **4** with or without mechanical agitation is identical (Figure 2c). Only after emergence of **1_6_** the formation of **4** starts to accelerate, demonstrating the catalytic effect of **1_6_** on the formation of **4**.

We compared the catalytic efficiency of **1_6_** to replicators with lysine‐to‐arginine mutations to assess the role of the lysine residues. Either the inner or the outer lysine was exchanged for arginine (yielding XGL**R**FK and XGLKF**R**, respectively) while still obtaining a hexamer replicator in both cases.^[^ ^35^ ^]^ Similarly to lysine, the side chain of arginine is positively charged yet it is a much poorer nucleophilic catalyst due to its higher p*K_a_*. Neither lysine to arginine mutation resulted in a large change in catalytic activity (Figure 2d), suggesting that both lysines are active.

To further support the catalytic role of the lysine sidechains, we subjected a mixture of **1_6_** and **2** to a reductive amination with sodium cyanoborohydride which selectively reduces imines to amines. Mass spectroscopy analysis confirmed the presence of reduced imine species, demonstrating that imines can form between **1_6_** and **2** (Figure 2f, Figure S14, Table S4). Using mass fragmentation and reduction of **1_6_** to **1** after the reductive amination, we were able to distinguish between inner and outer amine modification and determined a ratio of 1: 1.31 (± 0.02) of the outer to inner lysine (Figure S15, S16, Table S5). Subjecting monomer **1** to the same reductive amination procedure hardly resulted in any modification (Figure S15a) highlighting the importance of replicator assembly for imine formation.

Catalysis of hydrazone formation is likely to require the presence of non‐protonated lysines in the replicators. Lysine molecules, when dissolved in water, would be fully protonated at the pH of the experiments, so some shift in the protonation state needs to have occurred to make the self‐assembled replicator fibers catalytically active. This can, in principle, be due to the fibers offering a different local pH compared to bulk solution, or due to a shift in the p*K_a_* of the lysines upon assembly. To assess the pH at the fiber surface a pH indicator (bromothymol blue) was added to a solution of **1_6_** in borate buffer (50 mM, pH 8.2). The hydrophobic surface and negative charge of this indicator are expected to aid binding to the replicator fibers.^[^ ^60^ ^]^ The dye reported a local pH that was about 0.3 pH units lower than the pH of the buffer, which may indicate that the local pH is similar to that of the bulk solution (Figure S12). But note that the dye may not report on the exact sites where catalysis takes place and that interpretation is further complicated by possible effects of the polarity of the local microenvironment on the ionization equilibrium of the dye. We then probed the apparent p*K_a_* of the catalytically active lysines by monitoring the kinetics of acyl hydrazone formation catalyzed by **1_6_** as a function of pH. The resulting pH‐rate profile displayed a single inflection point at a pH of 8.31 ± 0.02 (Figure 2e). Thus, the apparent p*K_a_* of the active lysine residues is shifted by about 2 units compared to lysine in water. In earlier work we performed a pH titration of **1_6_** replicator fibers in water.^[^ ^35^ ^]^ This data showed no clear plateau around the determined apparent p*K_a_* value, indicating that the catalytically active lysines represent only a small fraction of all lysines.

To further characterize catalysis by replicator **1_6_**, we conducted saturation experiments for both substrates **2** (10 – 400 µM) and **3** (0.10 – 30 mM) at a single fixed concentration of the other substrate (**3**: 0.40 mM; **2**: 80 µM, respectively, Figure S10). Assuming standard Michaelis‐Menten kinetics, both **2** (*K_m,app,2_* = 37 ± 5 µM) and **3** (*K_m,app,3_* = 8 ± 2 mM) showed enzyme‐like saturation indicating that substrate binding microenvironments are present on the (probably not well‐ordered) fiber structure^[^ ^61^ ^]^ that facilitate catalysis. The benefit of introducing favorable electrostatic interactions for substrate recruitment is reflected by the low *K_m,app_* value for **2**. The *K_m,app_* value for **3** is considerably larger and less specific interactions are expected for its interaction with **1_6_**.^[^ ^62^ ^]^

Care has to be taken when further deriving kinetic parameters as unlike in enzymes, the number of catalytic sites on the replicator fibers is a priori unknown. If we assume each building block to represent one catalytic site an apparent turnover number (*k_cat,app_*) can be estimated of 2 x10^−3^ s^−1^ (by measuring the initial rate at substrate concentrations about a tenfold or more of their respective *K_m,app_* values). With this, values of the apparent third order constant (*k_cat,app_*/(*K_m,app,2_K_m,app,3_*)) of 9 x10^3^ M^−2^ s^−1^, the apparent chemical proficiency (1/*K_TS,app_* = *k_cat,app_*/(*K_m,app,2_K_m,app,3_*)/*k_uncat_*) of 6 x10^6^ M^−1^ and the effective molarity (*k_cat,app_*/*k_uncat_*) of 2 M (Table S3) could also be estimated. These kinetic parameters are comparable to (computational) designer enzymes for other bimolecular reactions,^[^ ^63^, ^64^, ^65^, ^66^, ^67^, ^68^, ^69^ ^]^ including an enzyme designed for acyl hydrazone formation (Table S6).^[^ ^52^, ^70^ ^]^

Note that in the above analysis we assumed all building blocks to be active in catalysis. However, the number of catalytically active lysines is likely smaller. In previous work on retro‐aldol catalysis (also thought to proceed through an iminium intermediate) by the same replicator we have conservatively estimated that less than 10% of lysines, (or less than 20% of building blocks) are catalytically active.^[^ ^35^ ^]^ If the fraction would be similar for the hydrazone catalysis, the kinetic parameters derived above would underestimate the actual catalytic activity of the replicator.

To study the scope and limitations of the reaction we expanded the substrates to include positively and negatively charged ones, as well as aliphatic and aromatic substrates, screening a total of seventeen reactions. We monitored the reactions by UV–vis spectrophotometry and calculated the ratio of the initial rates in the absence and presence of replicator **1**_6_ and we confirmed product formation by UPLC and mass spectrometry (Figure S17).

In general, aromatic substrates outperformed aliphatic ones and the presence of a positive charge (quaternary ammonium) was detrimental for catalysis. The best results were obtained when a negative charge (sulfonate or carboxylate) was present on the aldehyde. However to our surprise, the presence of a double negative charge on both the aldehyde and hydrazide did not improve or even reduced catalysis (Figure 3).

**Figure 3:**
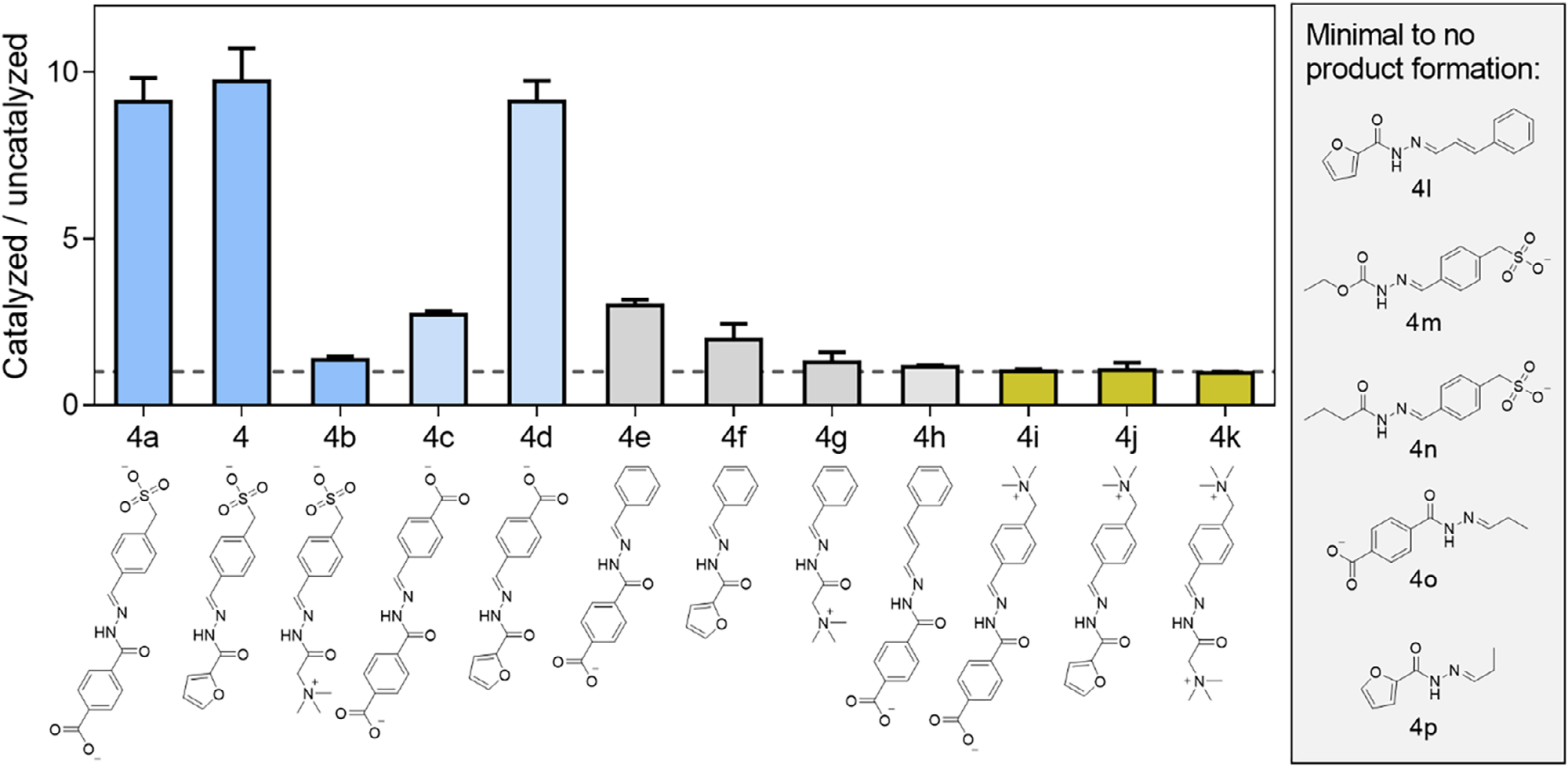
Substrate scope of acyl hydrazone formation catalyzed by **1_6_** (25 µM) from various aldehydes (50 µM) and hydrazides (200 µM) in borate buffer (pH 8.2). Initial rates were measured spectrophotometrically and divided by the initial rate without catalyst. Measurements were conducted as independent triplicates and error bars represent a single standard deviation. The dashed line indicates a ratio of catalyzed to uncatalyzed reaction of 1 (no catalysis).

The catalytic promiscuity of the replicator is remarkable: the same self‐replicator was previously reported to catalyze retro‐aldol cleavage as well (with an efficiency comparable to the best computationally designed enzymes for this reaction), while it is also an efficient catalyst for FMOC cleavage and promotes cofactor‐mediated photoredox catalysis.^[^ ^35^, ^36^ ^]^ Yet, the self‐replicator system was neither computationally designed or optimized nor evolved. In the evolution of enzymes, catalytic promiscuity is seen as an enabler for adaptation to changing environments and the development of more specialized enzymes, as it allows for one function to be repurposed as another (co‐option).^[^ ^71^, ^72^ ^]^ Promiscuity is also deemed important for early metabolic networks,^[^ ^73^ ^]^ and the fact that the self‐replicators exhibit this property is very promising for their further development in the direction of increasingly life‐like systems. More specifically, the ability of the β‐sheet assemblies to promote hydrazone bond formation holds considerable potential for constructing proto‐metabolic systems, that catalyze the formation of their own components from precursors in solution.

In conclusion, we expanded the catalytic abilities of our self‐replicating molecules from previously reported organocatalysis of bond‐breaking reactions, to evolutionary more relevant exogenous bond‐forming reactions. Specifically, the same self‐replicator that was previously found to catalyze a retro‐aldol reaction and FMOC cleavage, was now found to catalyze acyl hydrazone formation from aldehyde and hydrazide starting materials. We developed a well water‐soluble substrate/product pair for acyl hydrazone formation that is readily quantifiable by UV–vis spectrophotometry. The catalytic activity of the self‐replicator outperforms any of the replicator precursors and comes about through p*K_a_* perturbation of lysine residues in the replicator fibers. Furthermore, catalysis is an emergent property of the system, as the onset of replication coincides with an acceleration in product formation. The conservatively estimated kinetic parameters for this reaction compare favorably to other computationally designed enzymes for bimolecular reactions and various acyl hydrazone reactions can be catalyzed, especially when a negative charge is present. The catalytic promiscuity of this self‐replicator for both bond‐forming and bond‐breaking reactions and its ability to perform all of these reactions with high efficiency is remarkable and a promising starting point for further development of these systems through Darwinian evolution towards a minimal form of life.

## Supporting Information

The authors have cited additional references within the Supporting Information.^[^ ^74^, ^75^, ^76^, ^77^, ^78^, ^79^ ^]^

## Conflict of Interests

The authors declare no conflict of interest.

## Supporting information



Supporting Information

## Data Availability

The data that support the findings of this study are available from the corresponding author upon reasonable request.
