# Supplementary material for: Building Molecules by a Self‐Replicator That Catalyzes Acyl Hydrazone Formation
Source: Angew Chem Int Ed Engl. 2026 Jan 24;65(10):e06986. doi: 10.1002/anie.202506986 (PMC12955535; doi:10.1002/anie.202506986)
Supplement: Supplementary file 1 — Supporting Information [file ANIE-65-e06986-s001.pdf]

Supplementary Information

for

"Building Molecules by a Self-Replicator that Catalyzes Acyl Hydrazone Formation"

K. S. van Esterik, T. Marchetti and S. Otto

Table of Contents

1a. General methods 2
1b. Analytical methods 2
2a. Preparation of 1_6 4
2b. Preparation of $(XGLKFR)_6$, $(XGLRFK)_6$ and 1_6 4
2c. Preparation of $1_{3/4}$ 4
3a. Calibration of acyl hydrazone product 4 4
3b. Calibration of building block 1 on UPLC 5
4. Catalytic measurement of acyl hydrazone formation 5
5. Emergent catalysis of acyl hydrazone formation 5
6. Saturation experiments 6
7. Measurement of V_{max} 6
8. Comparison of initial rates of replicators with lysine-to-arginine mutations 7
9. Substrate scope 7
10. pH titration curve and pK_a determination 7
11. Indication of local pH 8
12. Probing turnover at a reduced concentration of 1_6 and TEM images 8
13. Detection of imine formation by reductive amination 8
14. Synthetic procedures 10
Sodium (4-formylphenyl)methanesulfonate (2) 10
Sodium (E)-(4-((2-(furan-2-carbonyl)hydrazineylidene)methyl)phenyl)methanesulfonate (4) 10
(4-formylbenzyl)trimethylammonium bromide 10
16. Supplementary Data 11
17. Reference Tables 30
18. References 31

1a. General methods

Building block **1** was purchased from Cambridge Peptides (Birmingham) as the trifluoroacetate salt after supplying 3,5-bis(tritylthio)benzoic acid, which was synthesized using literature procedures.[1] 4-(Bromomethyl)benzaldehyde was purchased from BLDpharm & Fluorochem, 2-furoic hydrazide, benzaldehyde, 4-formylbenzoic acid, cinnamaldehyde, propionaldehyde, Girard reagent T, ethylcarbazate and sodium sulfite were purchased from Sigma-Aldrich. Sodium perborate tetrahydrate was purchased from Merck, butyrohydrazide was purchased from TCI and 4-(hydrazinocarbonyl)benzoic acid was purchased from Apollo Scientific. Water, acetonitrile and trifluoroacetic acid of ultra liquid chromatography / mass spectrometry (ULC/MS) grade were obtained from Biosolve. Reverse phase flash chromatography was conducted on a Grace Reveleris X2 using high performance liquid chromatography (HPLC) grade acetonitrile from Honeywell (Riedel-de Haën) and MilliQ water. Borate buffers were prepared from boric anhydride (B_2O_3, >99.98% trace metal basis, Sigma Aldrich) in ULC/MS grade water or in doubly distilled water from an in-house double distillation set up, and the pH was adjusted to 8.2 with a 5% or 10% w/w solution of sodium hydroxide. All other chemicals were obtained from commercial suppliers and all chemicals were used without further purification. All concentrations of $\mathbf{1}_6$, $\mathbf{1}_{3/4}$ and **1** are expressed in concentration of building block **1**. Borate buffer concentrations are expressed in concentration of boron atoms.

1b. Analytical methods

Spectrophotometric measurements were conducted on a JASCO V-660 or JASCO V-650 spectrophotometer, equipped with a Julabo F 12 cooling unit with a Julaba EO temperature controller set at 25°C using quartz cuvettes (Helma) with a pathlength of 1 cm or 0.1 cm. Measurements shown in Figure 3, S1E, S7, S9 and S12 were conducted on a JASCO V-650 spectrophotometer equipped with a JASCO PAC-743 peltier thermostatted temperature control set to 25°C.

Ultra performance liquid chromatography (UPLC) analysis was conducted on a Waters Aquity UPLC-H-class or H-class PLUS system equipped with a PDA detector. All measurements were conducted using a reverse phase column (Aeris Peptide 1.7 µm XB-C18 × 2.10 mm, Phenomenex) which was kept at 35°C during measurements. Samples were measured by injecting 5 or 10 µL. The temperature in the sample chamber was kept at 25°C unless mentioned otherwise. Sample separation was achieved with a flow rate of 0.3 mL/min and a gradient of water (+0.1% trifluoroacetic acid) and acetonitrile (+0.1% trifluoracetic acid) (Table S1). In a few cases formic acid (FA) was used as an eluent additive with an altered separation gradient (Table S2). When required, chromatograms were processed and integrated using ApexTrack integration in Waters Empower 3 software.

Positive electrospray ionization time-of-flight UPLC/ESI-TOF mass spectrometry measurements were conducted on a Waters Acquity UPLC-H-class system coupled with a Waters Xevo-G2 TOF and analyzed using Masslynx V4.1. Samples were measured by injecting 5 µL, using the same separation methods as for UPLC measurements (Table S1 and Table S2). The mass spectrometer was set to the following (default) parameters: capillary voltage 3.00 kV, source cone voltage 20 V, extraction cone voltage 4.0 V at a source temperature of 120°C, with a desolvation temperature at 450°C. Gas flow was set at 0 L/h for cone gas and 800 L/h for desolvation gas.

Negative electrospray ionization quadrupole time-of-flight UPLC/ESI-QTOF mass spectrometry measurements were conducted on a Waters Acquity UPLC-H-class system coupled with a Waters Xevo-G2 QTOF and analyzed using Masslynx V4.1. Samples were measured by injecting 5 µL, using a same separation method as for UPLC measurements (Table S1) with water and acetonitrile but with FA (+0.1%) as eluent additive to both solvents instead of TFA. The mass spectrometer was set to the following (default) parameters: capillary voltage 2.50 kV, source cone voltage 40 V, extraction cone voltage 4.0 V at a source temperature of 80°C, with a desolvation temperature at 150°C. Gas flow was set at 1 L/h for cone gas and 600 L/h for desolvation gas. Given the likelihood for changed retention times when using formic acid as a modifier

compared to trifluoroacetic acid in UPLC analysis, a chromatogram (diode array; 254 nm & 310 nm) was recorded that assigned the highest intensity of each recorded peak to either 254 or 310 nm. Mass spectrometry characterization of the by catalysis formed product peak **4** as observed by UPLC (highest intensity 310 nm) was in this way corroborated (Figure S6).

Mass fragmentation was performed using a Vanquish UPLC system coupled to an Orbitrap Exploris™ 480 Mass Spectrometer. Samples were measured by injecting 5 µL using the same column as for UPLC analysis with the separation method described in Table S2. Fragmentation was targeted at $\mathbf{1}_{mod\text{-}2}$ (m/z = 944.36), and was conducted following the (default) parameters: spray voltage, 3.50 kV; extraction cone voltage, 4.0 V; source temperature, 80°C; and desolvation temperature, 350°C. The gas flow was set at 1 L/h for the cone gas and 50 L/h for the desolvation gas. Fragmentation was performed using high-energy collision dissociation at a collision energy of 30%. The obtained data were analyzed using ThermoFisher Scientific Xcalibur and FreeStyle software.

High-resolution mass spectrometry (HRMS) was performed on a Orbitrap Exploris 480 with ESI negative ionization from a direct injection from methanol (+ 0.1% formic acid, 0.3 mL/min over 5 min).

Nuclear magnetic resonance (NMR) measurements were conducted on a Varian Mercury Plus 400 MHz (9.4 T) shielded magnet with a Varian 5 mm PFG AutoSW probe or on an Agilent MR 400 MHz (9.4 T) shielded magnet with a Varian 5 mm OneNMR probe. NMR spectra were processed using MestreNova x64.

Negative staining transmission electron microscopy was conducted on a Phillips CM120 operating at 120 kV. Samples of $\mathbf{1}_6$ (1.0 mM or 10 µM in borate buffer pH 8.2 (100 mM or 50 mM respectively)) were prepared by transferring a small aliquot (5 µL) on a 400 mesh copper grid covered with a thin carbon film (van Loenen instruments). After 1 minute incubation, the grid was blotted on filter paper to remove excess liquid. Then, the grid was stained twice with uranyl acetate (2 %) by depositing a saturated uranyl acetate solution (5 µL) on the grid, incubating for 30 seconds and blotting it on filter paper. Microscopy images were taken of the resulting grid and analyzed using ImageJ.[2]

Table S1: Gradient used for UPLC measurements with TFA as additive.

**Time (min)**	**% Water (+0.1% TFA)**	**% Acetonitrile (+0.1% TFA)**	**Curve**
Initial	90.0	10.0	Initial
1.00	90.0	10.0	6
1.30	75.0	25.0	6
3.00	72.0	28.0	6
11.00	60.0	40.0	5
11.50	5.0	95.0	6
12.00	5.0	95.0	6
12.50	90.0	10.0	6
17.00	90.0	10.0	6

Table S2: Gradient used for UPLC measurements with FA as additive.

**Time (min)**	**% Water (+0.1% FA)**	**% Acetonitrile (+0.1% FA)**	**Curve**
Initial	90.0	10.0	Initial
1.00	90.0	10.0	6
1.30	75.0	25.0	6
3.00	72.0	28.0	6
11.00	60.0	40.0	6
11.50	5.0	95.0	6
12.00	5.0	95.0	6
12.50	90.0	10.0	6
17.00	90.0	10.0	6

2a. Preparation of 1_6

Inside a 1.5 mL vial (Phenomenex, 12x32 mm) with PFTE/silicone screw cap, building block **1** (≥ 1.00 mg) was dissolved in borate buffer (100 mM, pH 8.2) and briefly vortexed to yield a 1.0 mM solution. If necessary, some solution was removed so that the final volume was about 1 mL. A magnetic stirring bar (2x7 mm, VWR) was added and the solution was stirred at 1200 rpm at 40°C using a IKA C-MAG HS 7 control stirring plate equipped with Thermo Fisher Compact Digital Dry Bath/Block heater with an HPLC vial suited metal adaptor. The mixtures were analyzed by UPLC (0.20 mM or 0.10 mM, 10 µL injection volume) to confirm $\mathbf{1_6}$ formation >80%. They were kept at 40°C until use for measurements, which for catalytic measurements were started between day 6-7 and day 21 after dissolution of **1**. Figure S2A shows a representative UPLC chromatogram, Figure S4D shows the mass spectrum and Figure S5 shows TEM images of the final mixture.

2b. Preparation of $(XGLKFR)_6$, $(XGLRFK)_6$ and 1_6

To accelerate replicator formation, a library of $(XGLKFR)_6$, $(XGLRFK)_6$ and $\mathbf{1_6}$ for the catalysis experiments in Figure 2d were prepared by mixing a solution of building block (≥ 1.00 mg) in borate buffer (100 mM, pH 8.2) with a solution of sodium perborate (0.7 eq, in 100 mM borate buffer pH 8.2) and incubating the resulting solution (2.0 mM) at room temperature for at least two hours. Next, an existing sample dominated by $\mathbf{1_6}$ was added as seed (5 mol% in the final solution) and the libraries were diluted to obtain a final concentration of 1.0 mM expressed in all present building block. The libraries were stirred and kept at 40°C like in procedure 2a, but were used from day 3 onwards. Figure S2d,e show a representative UPLC chromatograms of $(XGLKFR)_6$ and $(XGLRFK)_6$ and Figure S3 shows the respective mass spectra.

2c. Preparation of $1_{3/4}$

According to a known procedure[3], a sample dominated by $\mathbf{1_{3/4}}$ was prepared by briefly mixing a solution of **1** (final concentration 1.0 mM) in borate buffer (100 mM, pH 8.2) with a solution of sodium perborate (0.95 eq, in 100 mM borate buffer pH 8.2) and incubating the resulting solution (1.0 mM) at room temperature for at least two hours. Figure S2C and Figure S4B,C show respectively a representative UPLC chromatogram and mass spectra of the final mixture.

3a. Calibration of acyl hydrazone product 4

Five samples of **4** (> 2.00 mg) were weighed out and dissolved in appropriate quantities of borate buffer (50 mM, pH 8.2) to obtain stock solutions of 2.0 mM. Solutions were subsequently diluted to obtain concentrations of 0.10 – 20 µM and measured spectrophotometrically at 310 nm (Figure S1B). For UPLC measurement, four samples between 0.50 – 50 µM were prepared from the obtained stock solutions of 2.0 mM and directly injected (5 µL injection volume) on UPLC using a gradient of water (+0.1% TFA) and acetonitrile (+0.1% TFA) (Table S1, Figure S1A) and analyzed by integration at 340 nm using ApexTrack integration with Waters Empower 3 software. All measurements were taken within 5 minutes of stock solution preparation. It should be noted that under the acidic conditions of UPLC, **4** shows signs of hydrolysis during measurement (Figure S6). Therefore, both the method (Table S1) and injection volume (5 µL) were kept the same for both calibration and experimental measurements, to minimize the error resulting from this.

3b. Calibration of building block 1 on UPLC

Four samples of **1** (> 2.00 mg) were weighed out and dissolved in appropriate quantities of borate buffer (50 mM, pH 8.2) to obtain stock solutions of 2.0 mM. Solutions were subsequently diluted to obtain concentrations of 50 - 300 μM and the samples, including a control without **1**, were measured (5 μL injection volume) by UPLC using a gradient of water (+0.1% TFA) and acetonitrile (+0.1% TFA) (Table S1) with water injections between measurements. Chromatograms were analyzed by obtaining the total peak area between 4 – 11 min (to include other ring sizes of **1** that form due to slight oxidation from the start, Figure S2B) at 254 nm by ApexTrack integration with Waters Empower 3 software (Figure S1C).

4. Catalytic measurement of acyl hydrazone formation

For a catalytic measurement as shown in Figure 2A, the following stock solutions were prepared: $\mathbf{1_6}$ (1.0 mM, in 100 mM borate buffer pH 8.2, prepared according to protocol above), **2** (4.0 mM, in 50 mM borate buffer pH 8.2) and **3** (4.0 mM in 50 mM borate buffer pH 8.2, diluted from 20 mM in ethanol). Samples were prepared from these stock solutions by combining appropriate amounts of $\mathbf{1_6}$ and **2** in borate buffer (50 mM, pH 8.2) in a quartz cuvette (1 cm pathlength). Then, the samples were incubated for at least 10 min at 25°C. Finally, **3** was added, resulting in a final solution (1.0 mL) with $\mathbf{1_6}$ (50 μM), **2** (50 μM) and **3** (0.20 mM) in borate buffer (50 mM, pH 8.2) with 1% v/v ethanol. The formation of **4** was followed spectrophotometrically at 310 nm. After measurement, the recorded data was zeroed after addition of all components and quantified using a calibration curve of five independently weighted samples of **4** (Figure S1B, $\Delta\varepsilon_{310} = 3.69 \cdot 10^4$ M^{-1} cm^{-1}). Dilution of samples with an extinction outside the calibration range did not change the absorbance non-linearly so the extinction coefficient was applied to the full kinetic data. Experiments with **1** and $\mathbf{1_{3/4}}$ or without catalyst were conducted using stock solutions of **1** and $\mathbf{1_{3/4}}$ (1.0 mM, in 100 mM borate buffer pH 8.2) or by adding borate buffer (50 mM, pH 8.2) instead of $\mathbf{1_6}$. Data represents two independent repeats (from independently weighted stocks of **1**, **2** and **3**).

5. Emergent catalysis of acyl hydrazone formation

For emergent catalysis measurements, the following stock solutions were prepared: **1** (2.0 mM in 50 mM borate buffer pH 8.2), **2** (4.0 mM in 50 mM borate buffer pH 8.2) and **3** (4.0 mM in 50 mM borate buffer pH 8.2, diluted from 20 mM in ethanol). Appropriate amounts of **2** and **3** were added to an HPLC vial (12x32 mm, Phenomenex) with borate buffer (50 mM, pH 8.2) preincubated at 40°C and further incubated inside a UPLC sample chamber at 40°C for 15 min. Then, just prior to UPLC injection, **1** was added to obtain final concentrations of **1** (0.20 mM), **2** (0.20 mM) and **3** (0.80 mM) in a total volume of 1.0 mL with 4% v/v ethanol. Using an in-house developed stirring device[4] and a magnetic stirring bar (7x2 mm, VWR) in the vials, samples were stirred continuously at 1200 rpm inside the UPLC sample chamber at 40°C to allow continuous direct UPLC injections (5 μL injection volume). Using identical conditions, samples were prepared without stirring bar, and with stirring bar but without **1**. The experiment was conducted in duplicate. Formation of **4** and $\mathbf{1_6}$ were analyzed at 340 nm and 254 nm, respectively, by ApexTrack integration with Waters Empower 3 software, and quantified using a calibration curve of five independently weighted samples of **4** (Figure S1A) and **1** (Figure S1C). It should be noted that under the acidic conditions of UPLC, **4** shows signs of hydrolysis during measurement (Figure S6). Therefore, both the method (Table S1) and injection volume (5 μL) were kept the same for both calibration and experimental measurements, to minimize the error resulting from this.

6. Saturation experiments

Saturation of 2

The following stock solutions were prepared: $\mathbf{1_6}$ (1.0 mM, in 100 mM borate buffer pH 8.2, prepared according to protocol above), **2** (4.0 mM, in 50 mM borate buffer pH 8.2) and **3** (4.0 mM in 50 mM borate buffer pH 8.2, diluted from 20 mM in ethanol). Samples were prepared with a fixed concentration of $\mathbf{1_6}$ (10 μM) and **3** (0.40 mM) and a varying concentration of **2** (10, 30, 50, 100, 200, 350 μM) by combining appropriate amounts of $\mathbf{1_6}$ and **2** in borate buffer (50 mM, pH 8.2) in a quartz cuvette (1 cm). The samples were incubated for at least 10 min at 25°C after which an appropriate amount of **3** was added to obtain the desired concentrations in a volume of 1.0 mL with 2% v/v ethanol. The formation of **4** was followed spectrophotometrically at 310 nm for at least 10 minutes. Control measurement without $\mathbf{1_6}$ catalyst were measured separately using the same stock solutions by adding borate buffer (50 mM, pH 8.2) instead of $\mathbf{1_6}$ and were measured for 5 minutes after 10 min incubation at 25°C. The recorded data were zeroed after addition of all components and quantified using a calibration curve of five independently weighted samples of **4** ($\Delta\varepsilon_{310} = 3.69 \cdot 10^4$ M^{-1} cm^{-1}, Figure S1B). The control measurements were subtracted from the respective measurements with $\mathbf{1_6}$ and initial rates were determined with a linear fit using 5 minutes of measurement data, starting after 4 minutes. Three independent repeats (from independently weighted stocks of **1**, **2** and **3**) were conducted and the apparent $K_{m,app,2}$ value was then determined through a non-linear Michaelis-Menten fit for the triplicate initial rates at each concentration of **2** using Graphpad Prism 6.01 software.

Saturation of 3

The following stock solutions were prepared: $\mathbf{1_6}$ (1.0 mM, in 100 mM borate buffer pH 8.2, prepared according to protocol above), **2** (4.0 mM, in 50 mM borate buffer pH 8.2) and **3** (400 mM in 50 mM borate buffer pH 8.2, diluted from 2 M in ethanol). Samples were then prepared with a fixed concentration of $\mathbf{1_6}$ (10 μM) and **2** (80 μM) and a varying concentration of **3** (2.0, 5.0, 10, 20, 30 mM) by combining appropriate amounts of $\mathbf{1_6}$ and **2** and in borate buffer (50 mM, pH 8.2) in a quartz cuvette (1 cm). The samples were incubated for at least 10 min at 25°C after which an appropriate amount of **3** and 20% ethanol in borate buffer (50 mM, pH 8.2) was added to obtain the desired concentrations in a volume of 1.0 mL with 2% v/v ethanol. The formation of **4** was followed spectrophotometrically at 310 nm for at least 5 minutes. Control measurements without $\mathbf{1_6}$ catalyst were measured separately using the same stock solutions by adding borate buffer (50 mM, pH 8.2) instead of $\mathbf{1_6}$. The recorded data was zeroed after addition of all components and quantified using a calibration curve of five independently weighted samples of **4** ($\Delta\varepsilon_{310} = 3.69 \cdot 10^4$ M^{-1} cm^{-1}, Figure S1B). The control measurements were subtracted from the respective measurements with $\mathbf{1_6}$ and initial rates were determined with a linear fit using 3 minutes of measurement data, starting after 1.5 minutes. Three independent repeats (from independently weighted stocks of **1**, **2** and **3**) were conducted and the apparent $K_{m,app,3}$ value was then determined through a non-linear Michaelis-Menten fit for the triplicate initial rates at each concentration of **3** using Graphpad Prism 6.01 software.

7. Measurement of V_{max}

The following stock solutions were prepared: $\mathbf{1_6}$ (1.0 mM, in 100 mM borate buffer pH 8.2, prepared according to protocol above), **2** (4.0 mM, in 50 mM borate buffer pH 8.2) and **3** (750 mM in 50 mM borate buffer pH 8.2, diluted from 3.75 M in ethanol). Samples were prepared from these stock solutions such that the final concentrations of **2** and **3** were at least about a tenfold of their respective $K_{m,\text{app}}$ values. To this end, $\mathbf{1_6}$ (7.00 μL) and **2** (43.8 μL) were combined in borate buffer (264 μL, 50 mM, pH 8.2) in a quartz cuvette (0.1 cm pathlength). Then, the samples were incubated for at least 15 min at 25°C. Finally **3** (35 μL) was added, resulting in a final solution (350 μL) with $\mathbf{1_6}$ (20 μM), **2** (0.50 mM) and **3** (75 mM) in borate buffer (50 mM, pH 8.2) with 2% v/v ethanol. Control measurements without $\mathbf{1_6}$ catalyst were measured separately using the same stock solutions by adding borate buffer (50 mM, pH 8.2) instead of $\mathbf{1_6}$ and were measured for 5 minutes after 10 min incubation at

25°C. The recorded data were zeroed after the addition of all components and quantified using a calibration curve of five independently weighted samples of **4** ($\Delta\varepsilon_{310}$ = 3.69·10^4 M^{-1} cm^{-1}, Figure S1B). The control measurements were subtracted from the respective measurements with $\mathbf{1_6}$ and initial rates were determined with a linear fit using 40 seconds of measurement data, starting after 20 seconds. The reported $V_{max,\ app}$ is the average of three independent repeats (from independently weighted stocks of **1**, **2** and **3**) (Figure S11).

8. Comparison of initial rates of replicators with lysine-to-arginine mutations

The following stock solutions were prepared: $\mathbf{1_6}$, (XGLKF**R**)$_6$ and (XGL**R**FK)$_6$ (1.0 mM, in 100 mM borate buffer pH 8.2, prepared according to protocol 2b; referred to as 'replicator'), **2** (4.0 mM, in 50 mM borate buffer pH 8.2) and **3** (4.0 mM in 50 mM borate buffer pH 8.2, diluted from 20 mM in ethanol). Samples were prepared from these stock solutions by combining appropriate amounts of hexamer replicator and **2** in borate buffer (50 mM, pH 8.2) in a quartz cuvette (1 cm pathlength). The samples were incubated for at least 10 min at 25°C. Finally, **3** was added, resulting in a final solution (1.0 mL) with replicator (25 µM), **2** (50 µM) and **3** (0.20 mM) in borate buffer (50 mM, pH 8.2) with 1% v/v ethanol. The formation of **4** was followed spectrophotometrically at 310 nm. After measurement, the recorded data was zeroed after addition of all components and quantified using a calibration curve of five independently weighted samples of **4** (Figure S1B, $\Delta\varepsilon_{310}$ = 3.69·10^4 M^{-1} cm^{-1}). Control measurements were conducted and processed in similar conditions without replicator present and were subtracted from the respective measurements. Data represents three replicates using the same stock solutions for each respective measurement.

9. Substrate scope

For the substrate scope shown in Figure 3, the following stock solutions were prepared from three independently weighted stocks: $\mathbf{1_6}$ (1.0 mM, in 100 mM borate buffer pH 8.2, prepared according to protocol 2a), aldehyde stock solutions (4.0 mM in 50 mM borate buffer pH 8.2 without cosolvent, except for cinnamaldehyde which was diluted from 20 mM in ethanol), hydrazide stock solutions (4.0 mM in 50 mM borate buffer, pH 8.2 without cosolvent except for furoic hydrazide (**3**), which was diluted from 20 mM in ethanol). In a typical experiment, the samples were prepared from these stock solutions by combining appropriate amounts of $\mathbf{1_6}$ and aldehyde in borate buffer (50 mM, pH 8.2) in a quartz cuvette (1 cm pathlength). Then, the samples were incubated for at least 10 min at 25°C. Finally, hydrazide was added, resulting in a final solution (1.0 mL) with $\mathbf{1_6}$ (25 µM), aldehyde (50 µM) and hydrazide (0.20 mM) in borate buffer (50 mM, pH 8.2) with 1% or 0.25 v/v ethanol when respectively hydrazide **3** or cinnamaldehyde was used. The formation of product was followed spectrophotometrically at 310 nm or 285 nm (for **4m** and **4n**). After measurement, the solutions were transferred to an HPLC vial (12x32 mm, Phenomenex), and product formation was confirmed via UPLC and mass spectroscopy. The recorded spectrophotometric data were zeroed after the addition of all components. The initial rates were determined with a linear fit using 30 minutes of measurement data and divided by control measurements. Data represents three independent repeats (from independently weighted stocks of **1**, aldehyde and hydrazide).

10. pH titration curve and pK_a determination

For the pH titration curve, the following stock solutions were prepared in triplicate: $\mathbf{1_6}$ (1.0 mM in 50 mM borate buffer pH 8.2, prepared according to protocol 2a), **2** (4.0 mM in 50 mM borate buffer pH 8.2) and **3** (4.0 mM in 50 mM borate buffer pH 8.2, diluted from 20 mM in ethanol). The buffer used for catalytic measurements was bis-trispropane (BTP), which has a buffering range between pH 6.3 and 9.5. In a typical experiment, the samples were prepared from the stock solutions by combining appropriate amounts of $\mathbf{1_6}$ and **2** in BTP buffer (50 mM, varying pH) in a quartz cuvette (1 cm pathlength). Then, the samples were incubated for at least 10 min at 25°C. Finally, **3** was added, resulting in a final solution (1.0 mL) with $\mathbf{1_6}$ (25 µM), **2** (50 µM) and **3** (0.20 mM) in BTP buffer (50 mM, at varying pH) with 1% v/v ethanol. An appropriate control without $\mathbf{1_6}$ was also

prepared following the same scheme. The formation of **4** was followed spectrophotometrically at 310 nm for at least 30 minutes and the formation of the product **4** was confirmed through UPLC. Data were fitted using equation (1) below using the Solver function in Excel. The reported apparent pK_a is the average of the three independent repeats and the reported error represents a single standard deviation.

$$V_{ini} = \frac{(V_{ini})_{max}}{1+10^{(pKa-pH)}} \quad (1)$$

11. Indication of local pH

Bromothymol Blue (BTB) was employed to assess the local pH on the replicator fiber surface. First, a calibration curve of BTB (10 µM) in bis-trispropane buffer at different pHs was made. Then, $\mathbf{1_6}$ (prepared according to protocol 2a, final concentration 50 µM) was added to a solution containing BTB (10 µM) in borate buffer (50 mM, pH 8.2). The comparison of the absorption spectra in the presence and absence of $\mathbf{1_6}$ showed a decrease of 0.06 units in the absorption maximum at 620 nm, corresponding to an apparent local pH of 7.9 (Figure S12). This result should be interpreted with caution as other factors, such as a more hydrophobic local environment may influence the protonation equilibrium of BTB.

12. Probing turnover at a reduced concentration of $\mathbf{1_6}$ and TEM images

For the reaction at a reduced concentration of $\mathbf{1_6}$ (Figure S7), the following stock solutions were prepared from three independently weighted stocks: $\mathbf{1_6}$ (1.0 mM, in 100 mM borate buffer pH 8.2, prepared according to protocol 2a), **2** (4.0 mM, in 50 mM borate buffer pH 8.2) and **3** (4.0 mM in 50 mM borate buffer pH 8.2, diluted from 20 mM in ethanol). Samples were prepared from these stock solutions by combining appropriate amounts of $\mathbf{1_6}$ and **2** in borate buffer (50 mM, pH 8.2) in a quartz cuvette (1 cm pathlength). Then, the samples were incubated for at least 10 min at 25°C. Finally, **3** was added, resulting in a final solution (1.0 mL) with $\mathbf{1_6}$ (10 µM), **2** (50 µM) and **3** (0.20 mM) in borate buffer (50 mM, pH 8.2) with 1% v/v ethanol. The formation of **4** was followed spectrophotometrically at 310 nm. Appropriate control experiments were conducted in triplicate without $\mathbf{1_6}$. After measurement, the recorded data were quantified using a calibration curve of five independently weighted samples of **4** (Figure S1B, $\Delta\varepsilon_{310} = 3.69 \cdot 10^4$ M^{-1} cm^{-1}). One of the catalytic samples with $\mathbf{1_6}$ was then transferred to an HPLC vial and used to take TEM images (Figure S7b,c). As a control, a solution containing $\mathbf{1_6}$ (final concentration 10 µM in borate buffer 50 mM, pH 8.2) without substrates **2** & **3** was prepared and left to stand for 5 days before taking TEM images.

13. Detection of imine formation by reductive amination

Initial reductive amination of a mixture of $\mathbf{1_6}$ *and excess* ***2***

To perform the experiment reported in Figure S14, the following stock solutions were prepared: $\mathbf{1_6}$ (1.0 mM, in 100 mM borate buffer pH 8.2, prepared according to protocol 2a), **2** (4.0 mM, in 50 mM borate buffer pH 8.2) and sodium cyanoborohydride (0.50 mM in ULC/MS grade water). A sample was prepared from these stock solutions by combining appropriate amounts of $\mathbf{1_6}$ and **2** in borate buffer (50 mM, pH 8.2) and incubated at room temperature (~20°C) for 20 minutes. Then, sodium cyanoborohydride was added, resulting in a solution (0.20 mL) with $\mathbf{1_6}$ (0.10 mM), **2** (1.0 mM) and sodium cyanoborohydride (5.0 mM) in borate buffer (50 mM, pH 8.2). The resulting solution was kept at 25°C for 16 hours and analyzed using UPLC/ESI(+)-TOF mass spectrometry (Figure S14 & Table S4).

Reductive amination of a mixture of $\mathbf{1_6}$ *and* ***2***

To perform the experiment reported in Figure S15 & S16, the following stock solutions were prepared: $\mathbf{1_6}$ (1.0 mM, in 100 mM borate buffer pH 8.2, prepared according to protocol 2a), **2** (4.0 mM, in 50 mM borate buffer pH 8.2) and sodium cyanoborohydride (0.50 M in ULC/MS grade water). A sample was prepared from these stock solutions by combining appropriate amounts of $\mathbf{1_6}$ and **2** in borate buffer (50 mM, pH 8.2) and incubated at room temperature (~20°C) for 20 minutes. Then, sodium cyanoborohydride was added, resulting in a solution (0.20 mL) with $\mathbf{1_6}$ (0.10 mM), **2** (0.20 mM) and sodium cyanoborohydride (5.0 mM) in borate buffer (50 mM,

pH 8.2). The resulting solution was kept at 25°C for 16 hours, then 1.0 µL of TCEP solution (0.40 M in 50 mM borate buffer, pH 8.2, final TCEP concentration 2.0 mM) was added to reduce $\mathbf{1_6}$ to **1**. The sample was incubated for another 4 hours at 25°C and analyzed using UPLC with the standard separation method (Table S1) and with an optimized separation method that separated the two peaks corresponding to a single lysine modification ($\mathbf{1_{mod\text{-}2}}$, inner or outer lysine modification) with formic acid as the eluent additive (Table S1, Figure S15c). UPLC/ESI(+)-TOF mass spectrometry using the same separation method confirmed lysine modification (Figure S15d,e). Mass fragmentation on the two peaks corresponding to $\mathbf{1_{mod\text{-}2}}$ (m/z = 944.36) was conducted to determine which peak corresponded to the inner or outer lysine (Figure S16, Table S5). Sample preparation was repeated six times using three independent stock solutions of aldehyde **2** and two independently prepared libraries of $\mathbf{1_6}$. The ratio of inner to outer lysine was determined by UPLC measurement (Table S2) using ApexTrack integration in Waters Empower 3 software, and the reported ratio is the average of all six samples. The reported error represents a single standard deviation.

The procedure was repeated in duplicate with **1** instead of $\mathbf{1_6}$, in this case no appreciable amount of modification was observed (Figure S15a).

14. Synthetic procedures

Sodium (4-formylphenyl)methanesulfonate (2)

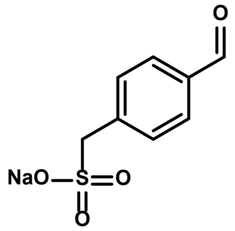

Synthetic procedure was derived from a literature procedure.[5]

Sodium sulfite (0.24 g, 1.9 mmol, 1.6 eq) was dissolved in water (2.4 mL) and 4-(bromomethyl)benzaldehyde (0.24 g, 1.2 mmol, 1.6 eq) was added to yield a suspension that clarified upon heating. The mixture was refluxed for 2 hours and cooled on ice. The resulting clear solution was filtered over a 0.2 µm filter and purified using reverse phase flash chromatography (C18, 40µm, 24g) with a gradient of water (MilliQ) / acetonitrile (HPLC grade) (without additives, 0-20%). Product fractions were lyophilized resulting in the desired white product (0.16 g, 0.72 mmol, 60%).

^{1}H-NMR (400 MHz, DMSO-d$_6$): δ 9.97 (s, 1H, COH), 7.80 (d, J = 8.2 Hz, 2H, CH), 7.51 (d, J = 8.2 Hz, 2H, CH), 3.82 (s, 2H, CH$_2$) (Figure S18). ^{13}C NMR (101 MHz, DMSO-d$_6$) δ 193.32, 143.23, 134.89, 131.37, 129.31, 58.03 (Figure S19). HRMS (ESI-neg): calculated for $C_8H_7O_4SNa$ [M-Na]$^-$: 199.0071, found: 199.0070.

Sodium (E)-(4-((2-(furan-2-carbonyl)hydrazineylidene)methyl)phenyl)methanesulfonate (4)

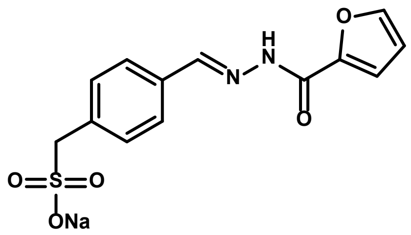

Furoic hydrazide (28 mg, 0.23 mmol, 1.0 eq) was added to sodium (4-formylphenyl)methanesulfonate (50 mg, 0.23 mmol, 1.0 eq) in methanol (1.5 mL). Then, 1 drop of acetic acid was added and the mixture was refluxed at 80°C for 3.8 hours. After the removal of methanol by reduced pressure, the crude reaction mixture was stored overnight in a freezer and then purified using reverse phase flash chromatography (C18, 40 µm, 24g) with a gradient of water (MilliQ) / acetonitrile (HPLC grade) (without additives, 3-20%). Product fractions were lyophilized resulting in the desired white product (54 mg, 0.16 mmol, 69%).

^{1}H NMR (400 MHz, D$_2$O) δ 8.22 (s, 1H), 7.83 – 7.62 (m, 3H), 7.43 (d, J = 8.2 Hz, 2H), 7.26 (dd, J = 3.6, 0.8 Hz, 1H), 6.65 (dd, J = 3.6, 1.8 Hz, 1H), 4.12 (s, 2H) (Figure S20). ^{13}C NMR (101 MHz, DMSO-d$_6$) δ 154.17, 148.14, 146.66, 145.81, 137.86, 132.16, 130.68, 126.38, 114.78, 112.04, 57.45 (Figure S21). HRMS (ESI-): calculated for $C_{13}H_{11}N_2NaO_5S$ [M-Na]$^-$: 307.0383, found: 307.0392.

(4-formylbenzyl)trimethylammonium bromide

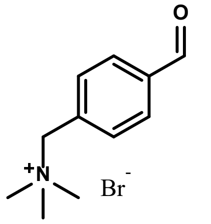

Synthetic procedure was derived from a literature procedure.[6]

4-Bromomethylbenzaldehyde (0.20 g, 1.0 mmol, 1.0 eq) of was added to 1.0 ml trimethylamine solution (2.6 mmol, 2.6 eq, 30 v/v% solution in ethanol) and the reaction mixture was stirred for three hours. The white precipitate that formed was filtered off and washed with small amounts of diethyl ether and dried under vacuum. The pure product was obtained as a white powder in 58% yield (0.15 g, 0.58 mmol).

^{1}H NMR (401 MHz, D$_2$O) δ 9.91 (s, 1H), 7.95 (d, J = 6.4 Hz, 2H), 7.66 (d, J = 8.2 Hz, 2H), 4.48 (s, 2H), 3.03 (s, 9H). (Figure S23) ^{13}C NMR (101 MHz, D$_2$O) δ 195.62, 137.09, 133.77, 133.55, 130.32, 68.76, 52.65, 52.61, 52.57 (Figure S24). HRMS (ESI-): calculated for $C_{11}H_{16}NO$ [M]$^+$: 178.123, found: 178.187.

16. Supplementary Data

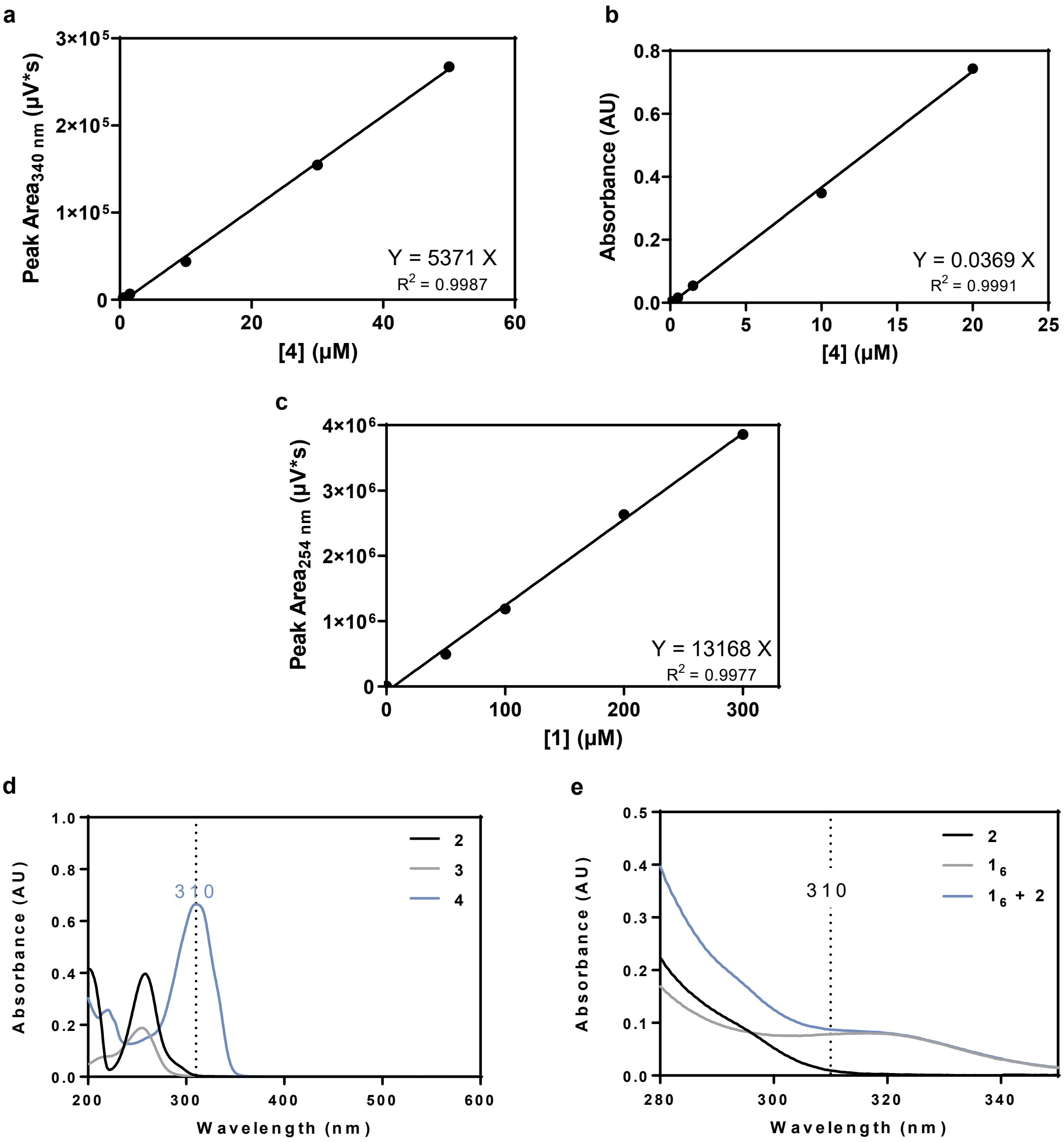

Figure S1: a) Calibration curve of **4** for UPLC at 340 nm. b) Calibration curve of **4** by UV-vis spectrophotometry at 310 nm. c) Calibration curve of **1** by UPLC at 254 nm. d) UV-vis spectrum of **2**, **3**, and **4** (20 µM in 50 mM borate buffer, pH 8.2) and maximum of **4**. e) UV-vis spectrum of $\mathbf{1}_6$, **2**, and $\mathbf{1}_6$ + **2** (each component 50 µM in 50 mM borate buffer pH 8.2, $\mathbf{1}_6$ diluted from a 1.0 mM stock solution in 100 mM borate buffer pH 8.2 prepared according to the protocol above) after incubation for at least 30 min at 20°C.

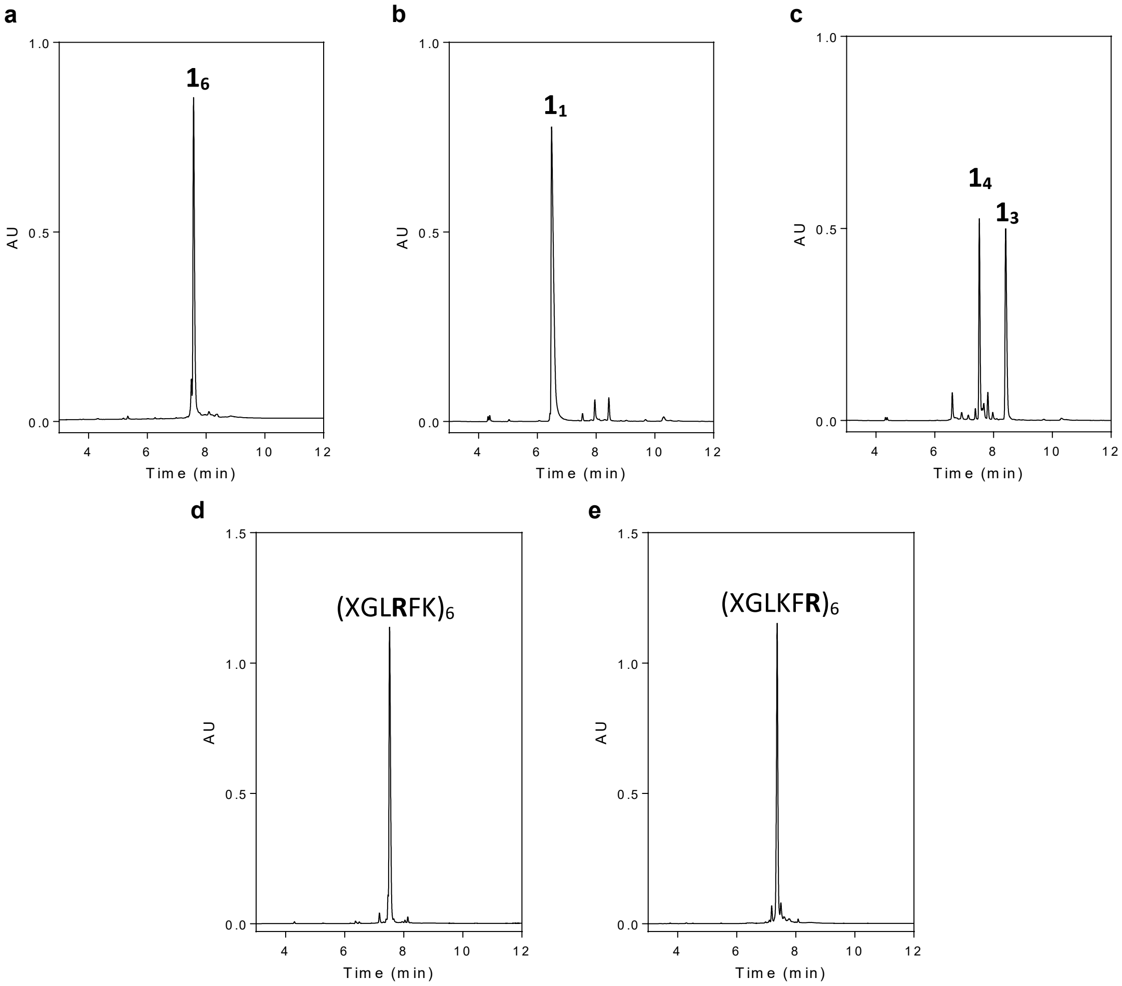

Figure S2: UPLC chromatograms at 254 nm of a) $\mathbf{1}_6$, b) $\mathbf{1}_1$, and c) $\mathbf{1}_{3/4}$, obtained by oxidation with sodium perborate as described in the methods section, d) (XGL**R**FK)$_6$, e) (XGLKF**R**)$_6$. To obtain (XGLKF**R**)$_6$ and (XGL**R**FK)$_6$ more quickly, they were prepared by pre-oxidation to 70% with sodium perborate and seeding with 5% $\mathbf{1}_6$.

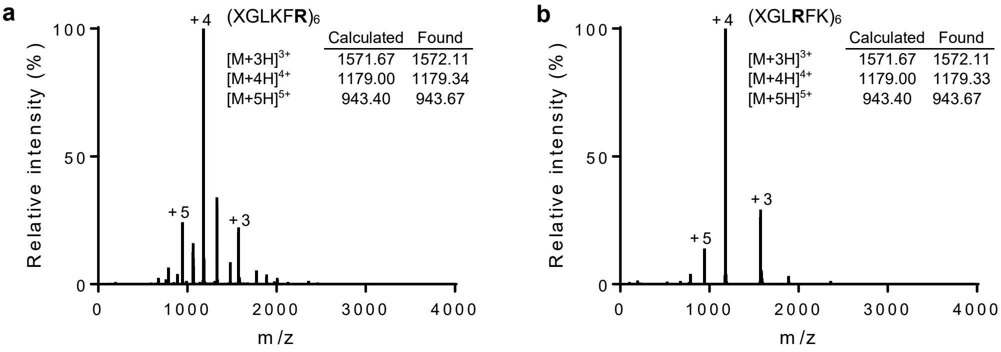

Figure S3: UPLC-MS (ESI+) of a) (XGLKF**R**)$_6$ and b) (XGL**R**FK)$_6$. Indicated are relevant charge states and calculated and found mass ions. To obtain (XGLKF**R**)$_6$ and (XGL**R**FK)$_6$ more quickly, they were prepared by pre-oxidation to 70% with sodium perborate and seeding with 5% $\mathbf{1}_6$.

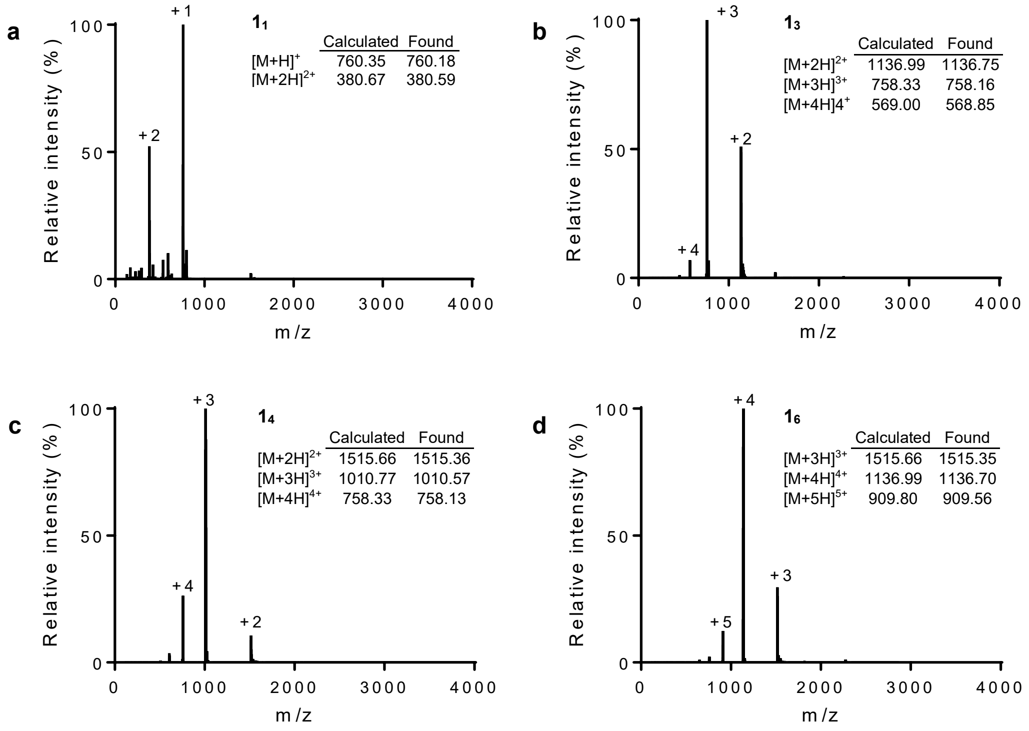

Figure S4: Representative UPLC-MS (ESI+) spectra of $\mathbf{1_1}$ (a), $\mathbf{1_3}$ (b), $\mathbf{1_4}$ (c) and $\mathbf{1_6}$ (d), indicated are relevant charge states and calculated and found mass ions.

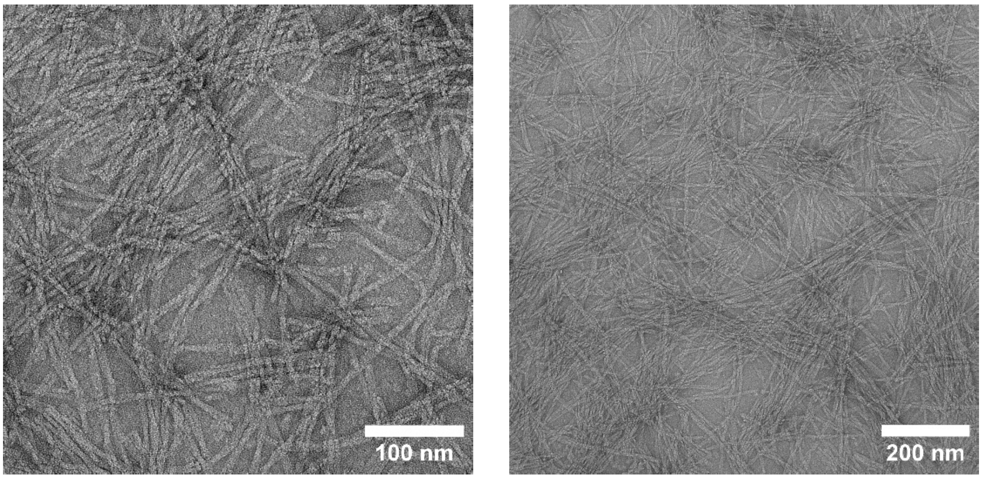

Figure S5: TEM images of $\mathbf{1_6}$ (1.0 mM) show the formation of fibers.

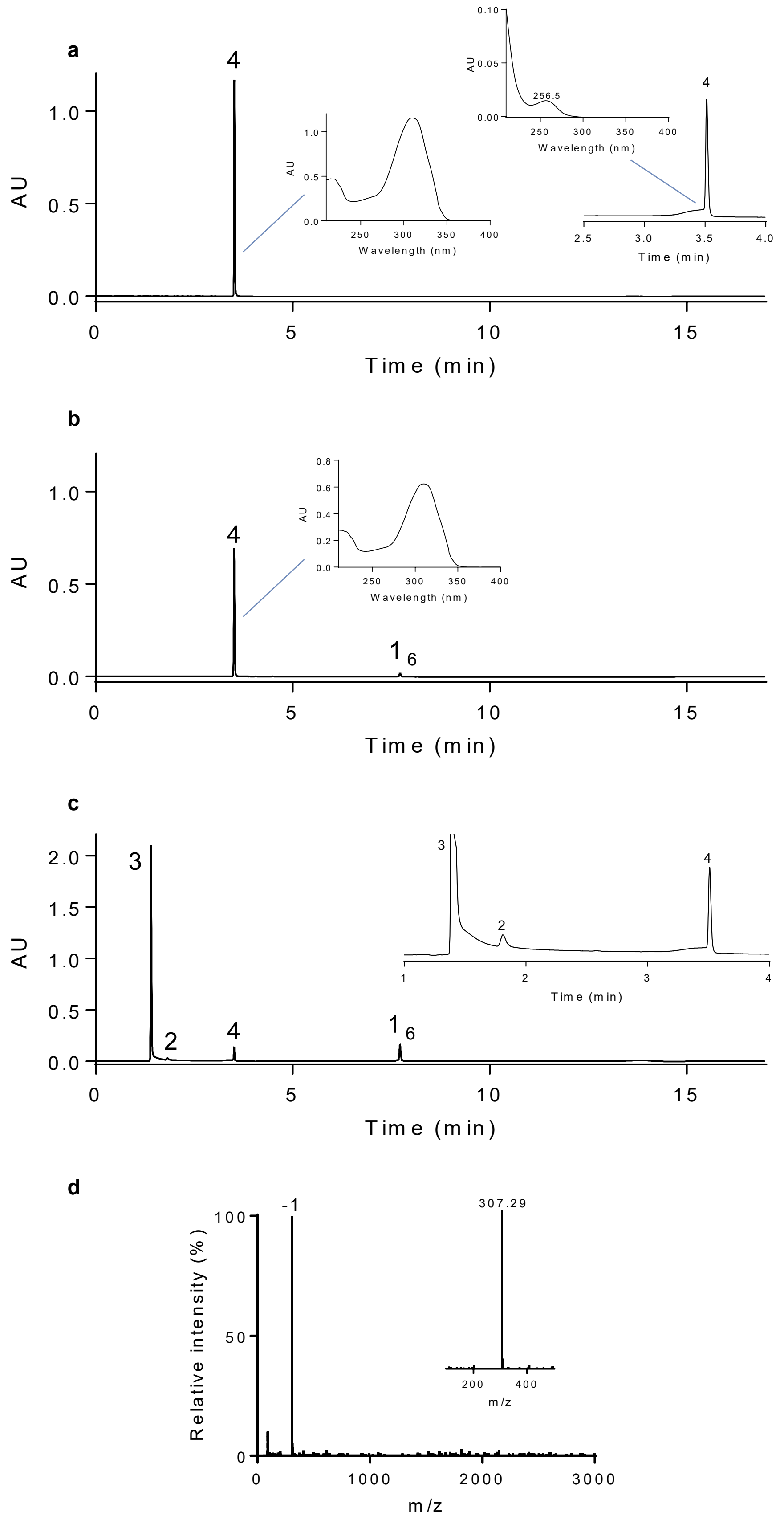
a
4
AU
1.0
0.5
0.0
0
5
10
15
Time (min)
Wavelength (nm)
256.5
Time (min)
b
4
1
6
AU
Time (min)
Wavelength (nm)
c
3
2
4
1
6
AU
2.0
1.5
1.0
0.5
0.0
Time (min)
d
-1
307.29
Relative intensity (%)
100
50
0
1000
2000
3000
m/z

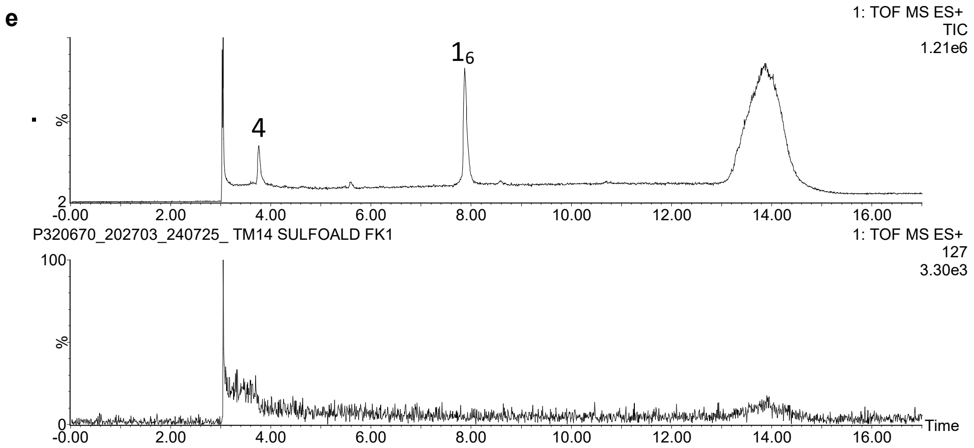

Figure S6 a) A UPLC chromatogram of reference **4** (50 µM) at 310 nm. Insets show the UV spectrum of the peak corresponding to **4**, the chromatogram at 254 nm between 2.5 and 4 minutes and the UV spectrum of a visible hump at 254 nm prior to the main peak of **4**. This hump hints at some hydrolysis, presumably caused by the 0.1% TFA eluent additive[7] (see also Figure S6E). The UV spectrum of the hump is similar to that of a combination of **2** and **3**. b) UPLC chromatogram at 310 nm of $\mathbf{1_6}$ (40 µM) incubated at 25°C with **2** (40 µM) and **3** (0.40 mM) after 1 day showing the formation of **4**. Inset shows the UV spectrum of the peak indicated as **4** which corresponds to that of the reference compound (Figure S6A and Figure S1D). c) The same UPLC chromatogram as in panel b, monitored at 254 nm, showing **3,** residual **2**, **4** and $\mathbf{1_6}$. Inset shows the chromatogram between 1-4 minutes, with a hump prior to peak **4** indicating some hydrolysis might occur in the acidic conditions during UPLC analysis (as in a). d) UPLC-MS (ESI-) mass spectrum of the same sample as b) and c) of peak **4**. Inset shows a zoomed in spectrum of the found mass. Calculated for **4** $[M\text{-}Na]^{-1}$: 307.04, found: 307.29. e) Top: UPLC-MS (ESI+) total ion count of a catalysis sample of **4** formation by $\mathbf{1_6}$. Bottom: UPLC-MS (ESI+) ion count of 127 corresponding to $[\mathbf{3}+H]^{+}$, showing an increased count fronting peak **4**. Mass detection was turned on after a cut off of 3 minutes, resulting in an initial spike in both panels.

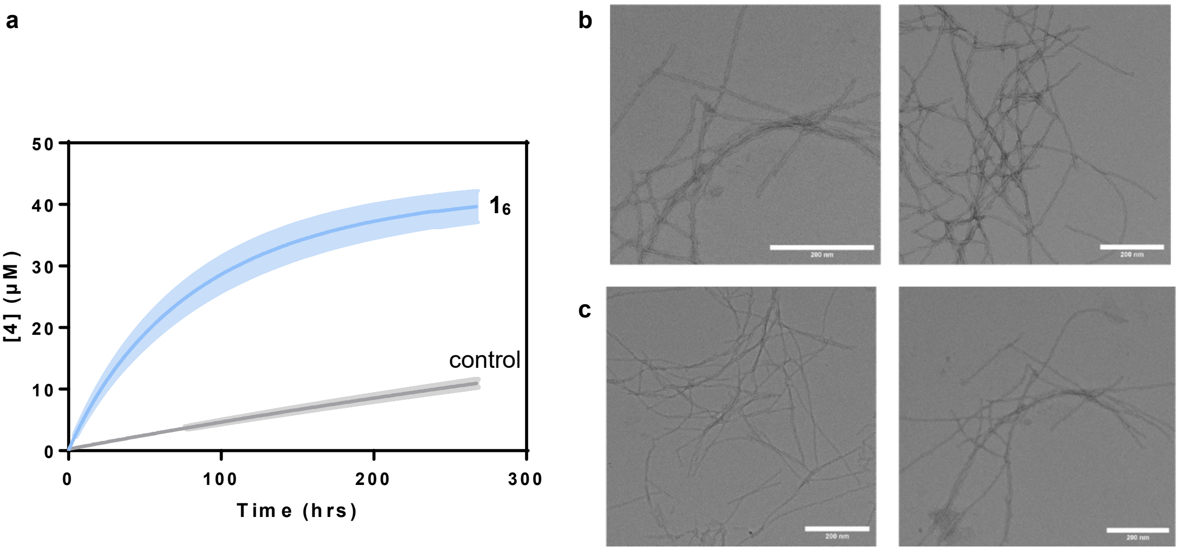

Figure S7: a) Formation of **4** from **2** (50 µM) and **3** (0.20 mM) in the presence of $\mathbf{1_6}$ (10 µM with respect to **1**, blue) and in the absence of $\mathbf{1_6}$ (grey). Measurements were conducted spectrophotometrically ($\Delta\varepsilon_{310} = 3.69 \cdot 10^{4}$ M^{-1} cm^{-1}) as independent triplicates and error bars represent a single standard deviation. b) TEM images of $\mathbf{1_6}$ (10 µM) after incubation of 5 days in borate buffer (50 mM, pH 8.2). c) TEM images of $\mathbf{1_6}$ (10 µM) after reaction of **2** and **3** as described in a).

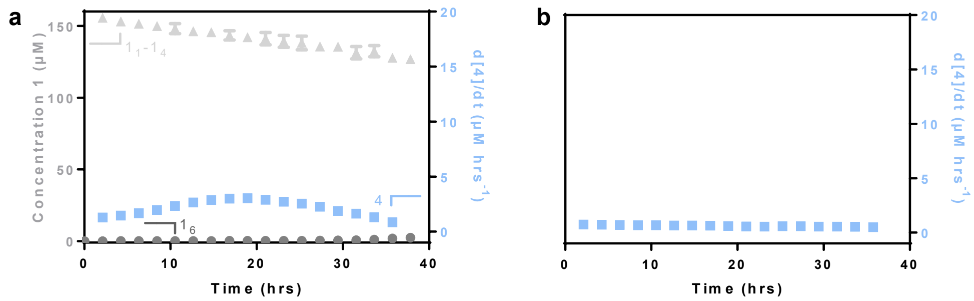

Figure S8: a) Control experiment for emergent catalysis of acyl hydrazone formation from **2** (0.20 mM) and **3** (0.80 mM) without stirring in presence of **1** (0.20 mM). b) Control experiment of acyl hydrazone formation from **2** (0.20 mM) and **3** (0.80 mM) in the absence of **1**.

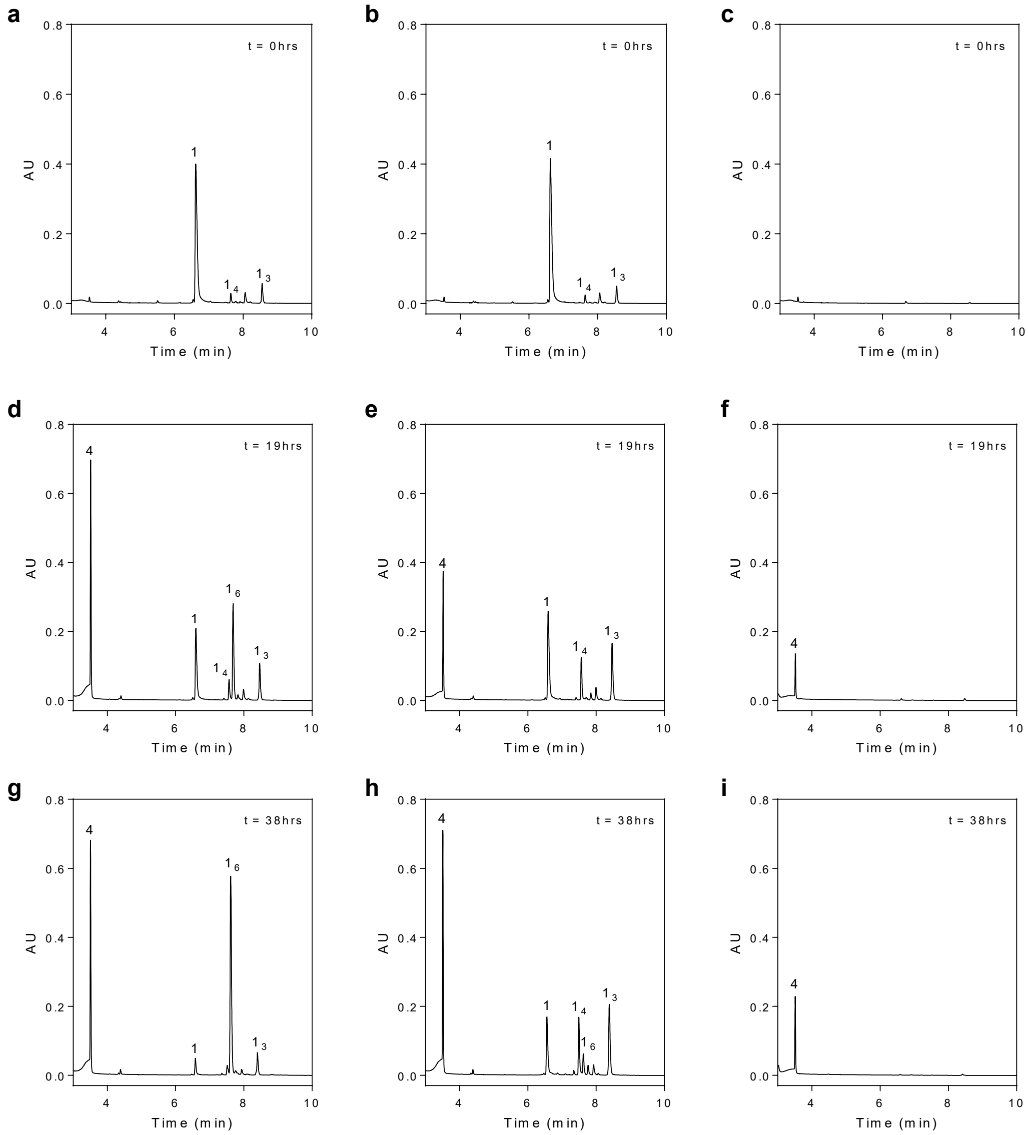

Figure S9: Representative chromatograms at the start, middle and end of the emergence experiment combined with acyl hydrazone catalysis shown in Figure 2b,c and Figure S8. From left to right: stirred sample with **1** (a, d, g), non-stirred control with **1** (b, e, h), control without presence of **1** (c, f, i).

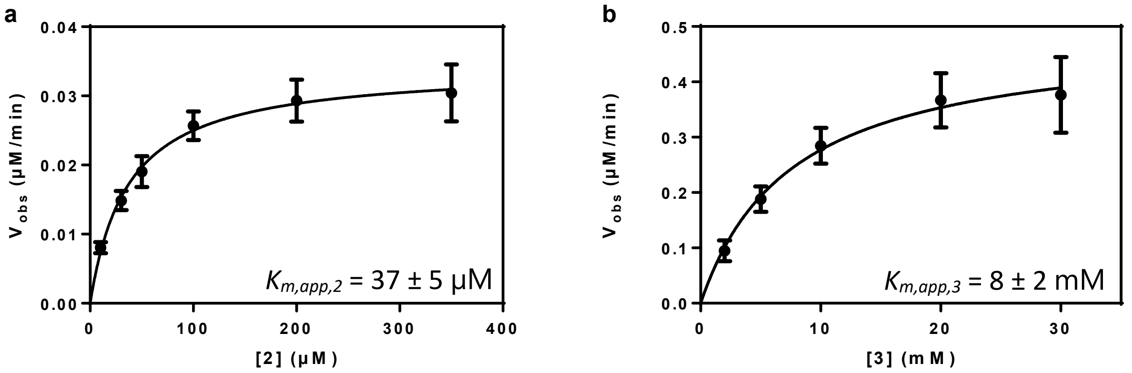

Figure S10: a) Observed initial rate of **4** formation in presence of $\mathbf{1_6}$ (10 µM) at a fixed concentration of **3** (0.40 mM) and a varying concentration of **2**. b) Observed initial rate of **4** formation in presence of $\mathbf{1_6}$ (10 µM) at a fixed concentration of **2** (0.40 mM) and a varying concentration of **3**. Data shows three independent repeats from weighing step (of **1**, **2**, and **3**) and error bars represent a single standard deviation.

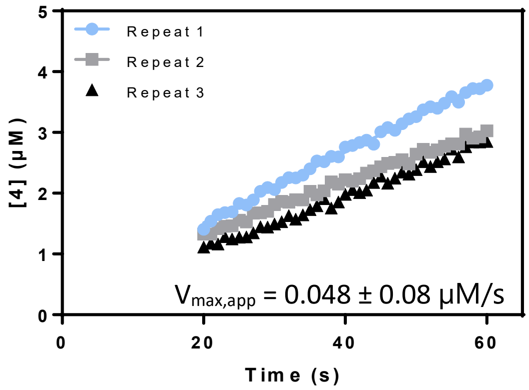

Figure S11: $V_{max,app}$ determination for $\mathbf{1_6}$ (20 µM) with **2** (0.50 mM) and **3** (75 mM) obtained by spectrophotometric measurement at 310 nm. Shown are three independent repeats (from the weighing step) after correction for control measurement without $\mathbf{1_6}$. The reported $V_{max,\,app}$ is obtained from the average of a linear fit for the three repeats. The reported error represents a single standard deviation.

Table S3: Estimated apparent kinetic parameters for acyl hydrazone formation by $\mathbf{1_6}$.

Name	k_{uncat}[a] (M^{-1} s^{-1})	$k_{cat,app}$[b] (s^{-1})	$K_{m,app,2}$[c] (M)	$K_{m,app,3}$[c] (M)	$k_{cat,app}$ / ($K_{m,app,2}$ $K_{m,app,3}$) ($M^{-2}s^{-1}$)	EM_{app}[d] (M)	$1/K_{TS,app}$[e] (M^{-1})
$\mathbf{1_6}$	(1.6 ±0.6) x10^{-3}	2 x 10^{-3}	(3.7 ±0.5) x10^{-5}	(8 ±2) x10^{-3}	9 x10^{3}	2	6 x10^{6}

[a] k_{uncat} was determined as the second order rate constant using the control measurements without replicator $\mathbf{1_6}$ of the saturation experiments of both **2** and **3**. The initial rate depended linearly on both substrates and k_{uncat} was obtained by averaging calculated apparent k_{uncat} values of the initial rates (triplicates) of all concentration pairs of **2** and **3**. [b] $k_{cat,app}$ was calculated by dividing $V_{max,app}$ (Figure S11) by the number of catalytic sites, which was conservatively estimated to correspond to the concentration of building block **1**. [c] $K_{m,app}$ values were obtained through a non-linear Michealis-Menten fit of the saturation data obtained in Figure S10. [d] Effective molarity (EM = k_{cat}/k_{uncat}), [e] chemical proficiency ($1/K_{TS}$ = [k_{cat}/($K_{m,S1}$ $K_{m,S2}$)]/k_{uncat}).

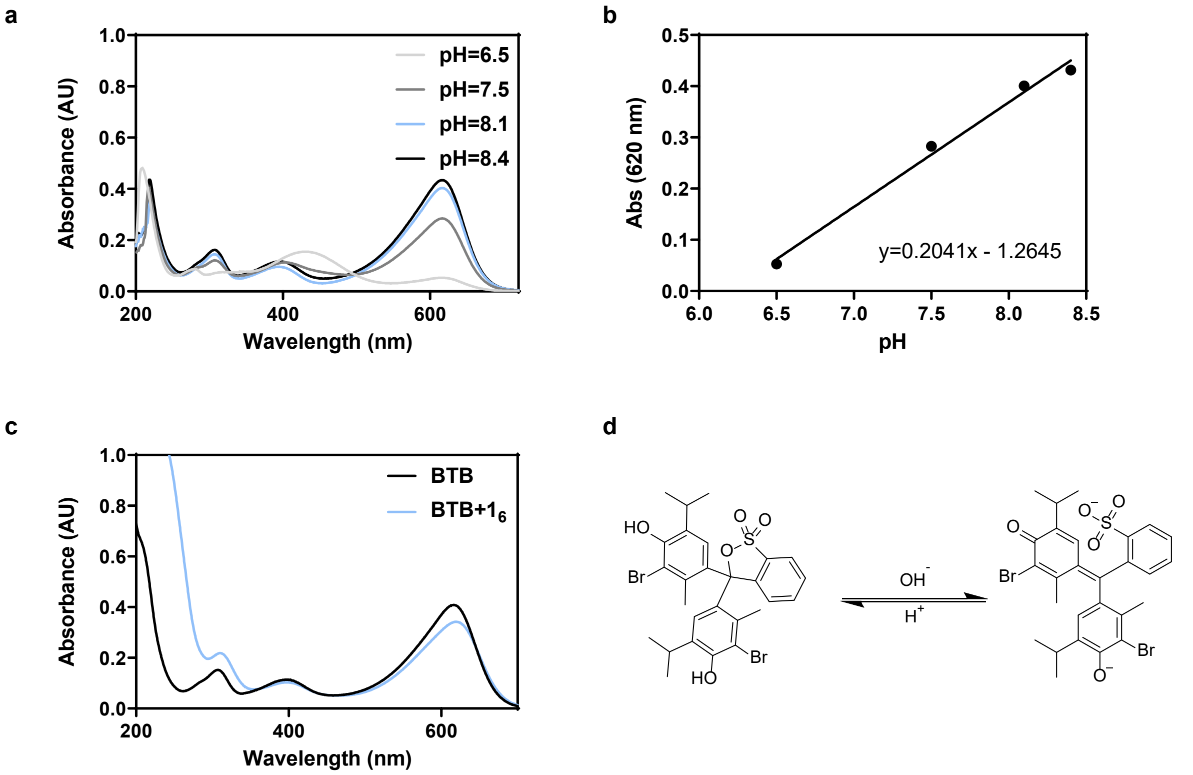

Figure S12: a) Absorption spectra of BTB (10 µM) at various pH values in bis-trispropane (BTP) buffer (50 mM, at 25°C). b) Absorption maximum of BTB at 618 nm as a function of pH. Note that in the narrow pH window that was probed a linear relationship between pH and absorbance was obtained, instead of the sigmoidal relationship that would be expected across a wider pH range based on to the Henderson-Hasselbalch equation. c) Absorption spectra of BTB (10 µM) in the presence (black line) and absence (light blue line) of **1**$_6$ (50 µM) in borate buffer (50 mM, pH 8.2, 25°C). d) Equilibrium between the closed and open forms of BTB.

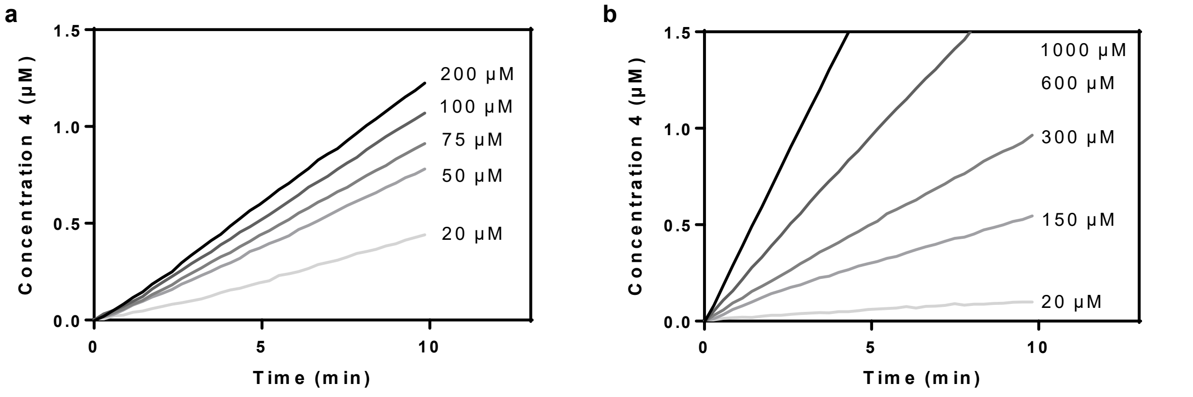

Figure S13: Initial formation of **4** catalyzed by **1**$_6$ (25 µM) in borate buffer (50 mM, pH 8.2) at 25 °C, with pre-incubation of **2** and **3** before addition of **1**$_6$, shows a very slight non-linear increase in product formation. Data was obtained spectrophotometrically at 310 nm and converted to concentration ($\Delta\varepsilon_{310} = 3.69 \cdot 10^4$ M^{-1} cm^{-1}) and a control measurement without catalyst was subtracted. a) Fixed concentration of **3** (0.40 mM) and varying concentration of **2** as indicated. b) Fixed concentration of **2** (0.40 mM) and varying concentration of **3** as indicated.

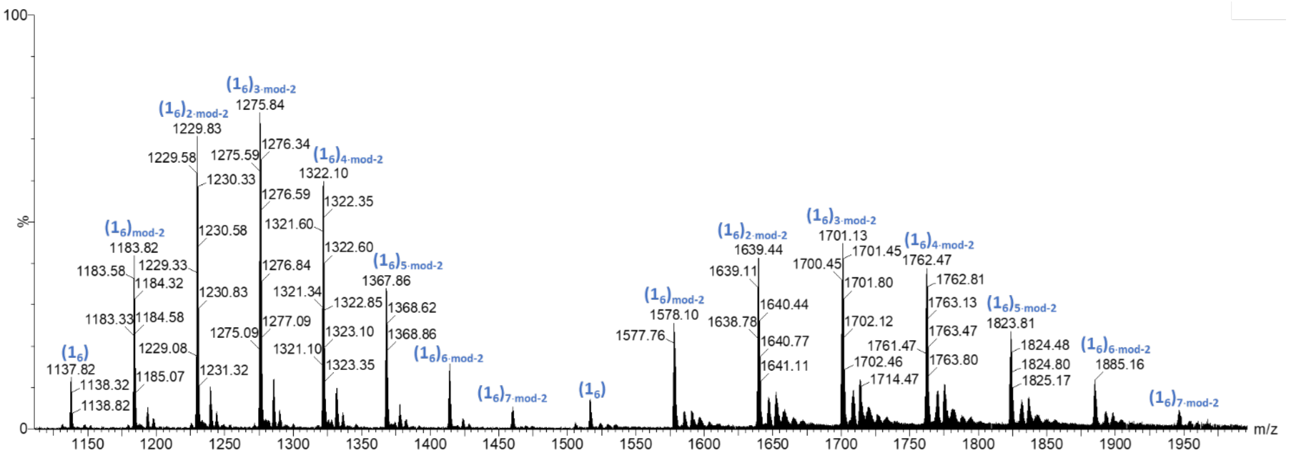

Figure S14: Partial mass spectrum of $\mathbf{1_6}$ (0.10 mM) modified with aldehyde **2** (1.0 mM) through reduction with sodium cyanoborohydride (5.0 mM) featuring up to seven reduced imine species per six-membered $\mathbf{1_6}$ ring. The mass values found and expected for the different species are reported in Table S4.

Table S4: Calculated and found mass ions for the mass spectrum in Figure S14.

Compound	Mass calculated for $[M+3H]^{3+}$	Mass found for $[M+3H]^{3+}$	Mass calculated for $[M+4H]^{4+}$	Mass found for $[M+4H]^{4+}$
(1_6)	1516.33	1516.75	1137.50	1137.82
$(1_6)_{mod-2}$	1577.34	1577.76	1183.26	1183.82
$(1_6)_{2\cdot mod-2}$	1639.35	1639.44	1229.76	1230.83
$(1_6)_{3\cdot mod-2}$	1700.35	1700.79	1275.52	1275.84
$(1_6)_{4\cdot mod-2}$	1761.69	1762.14	1321.52	1321.85
$(1_6)_{5\cdot mod-2}$	1823.70	1823.82	1367.53	1367.86
$(1_6)_{6\cdot mod-2}$	1884.37	1885.16	1413.53	1413.88
$(1_6)_{7\cdot mod-2}$	1945.71	1946.20	1459.54	1459.87

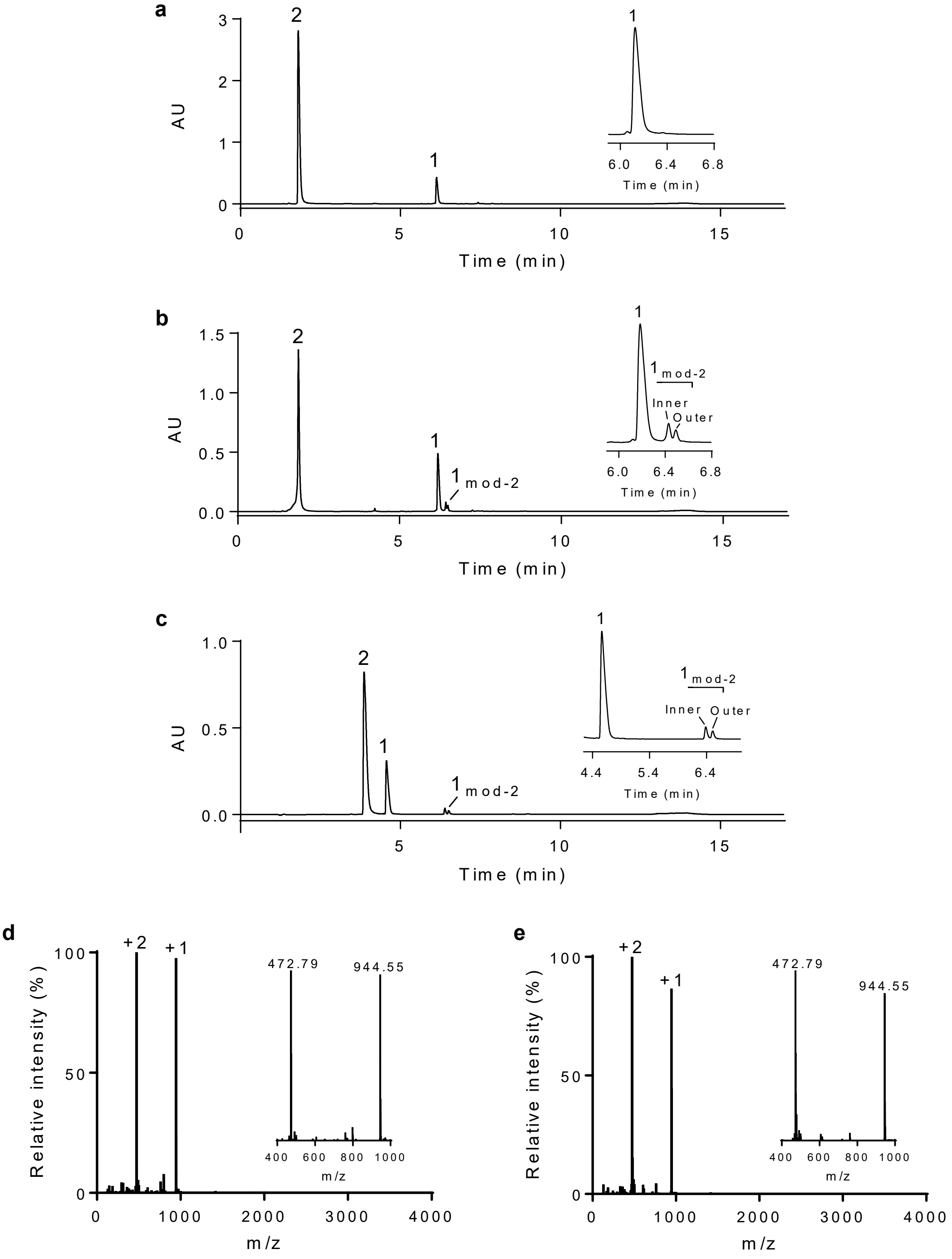

Figure S15: a) UPLC chromatogram (+0.1% TFA) of **1** (0.10 mM) after incubation with **2** (0.20 mM) and subsequent reductive amination with sodium cyanoborohydride (5.0 mM). Inset shows the chromatogram between 5.9 - 6.8 minutes. b) UPLC chromatogram (+0.1% TFA) of $\mathbf{1_6}$ (0.10 mM) after incubation with **2** (0.20 mM) and subsequent reductive amination with sodium cyanoborohydride (5.0 mM) and reduction to partially modified **1** with TCEP (2.0 mM). Reduced imine species are labeled $\mathbf{1_{mod\text{-}2}}$ and correspond to a single modification. Inset shows the chromatogram between 5.9 - 6.8 minutes. Assignment of inner and out lysine modification is based on results in Figure S16. c) UPLC chromatogram (+0.1 % FA) of the same sample as b) separated with an optimized gradient with formic acid (+0.1%) as eluent additive (Table S2). Reduced imine species are labeled $\mathbf{1_{mod\text{-}2}}$ and correspond to a single modification. Inset shows the chromatogram between 4.25 – 7.0 minutes. Assignment of inner and out lysine modification is based on results in Figure S16. d) UPLC-MS (ESI+) mass spectrum of the left peak of the peaks labeled as $\mathbf{1_{mod\text{-}2}}$ in c). Inset shows a zoomed in spectrum of the found masses. Calculated for $\mathbf{1_{mod\text{-}2}}$ $[M+2H]^{2+}$: 472.68, found: 472.79. Calculated for $\mathbf{1_{mod\text{-}2}}$ $[M+H]^{+}$: 944.36, found: 944.55. e) UPLC-MS (ESI+) mass spectrum of the right-most peak of the peaks labeled as $\mathbf{1_{mod\text{-}2}}$ in c). Inset shows a zoomed in spectrum of the found masses. Calculated for $\mathbf{1_{mod\text{-}2}}$ $[M+2H]^{2+}$: 472.68, found: 472.79. Calculated for $\mathbf{1_{mod\text{-}2}}$ $[M+H]^{+}$: 944.36, found: 944.55.

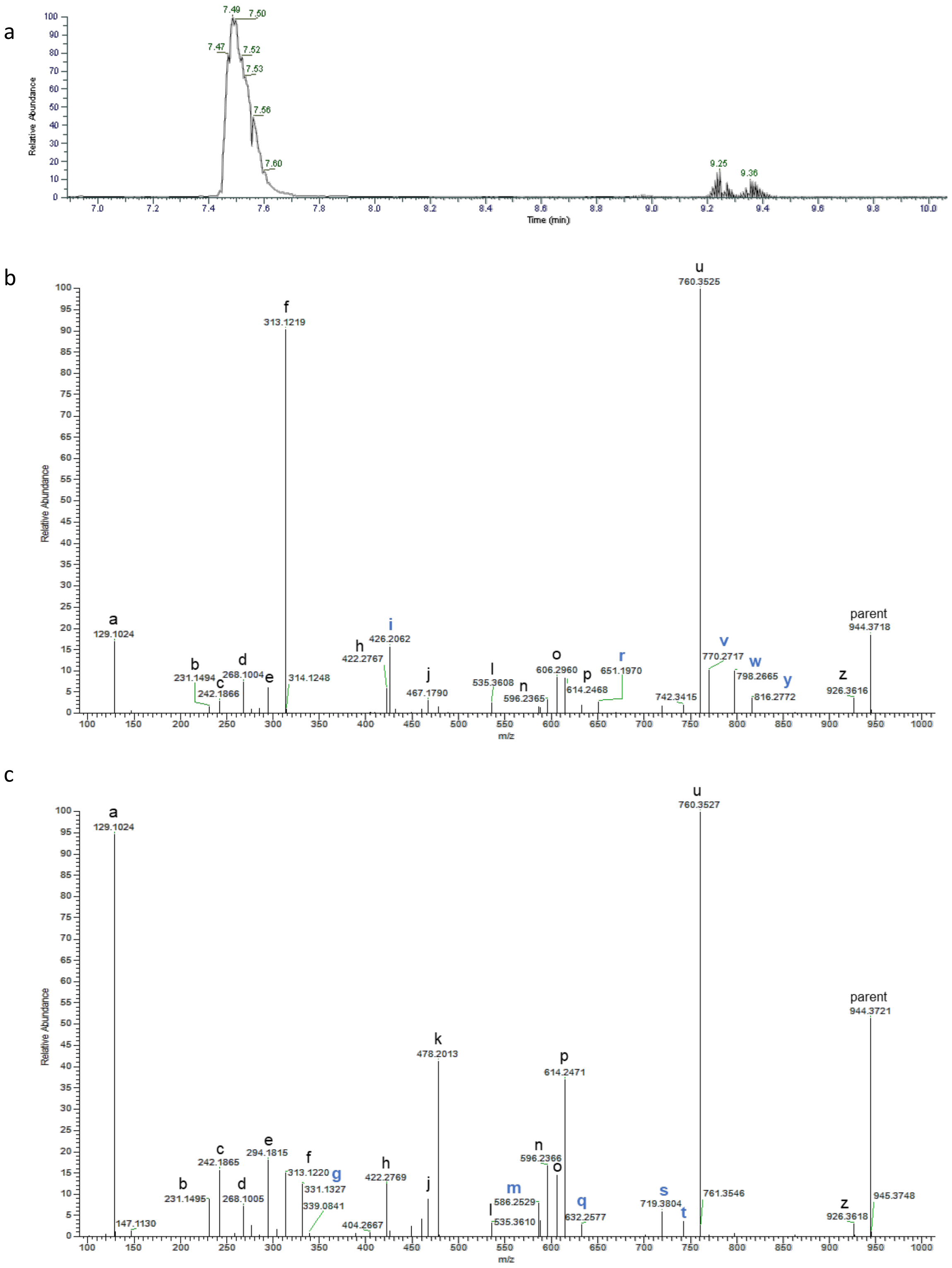

Figure S16: a) UPLC-MS (ESI+) total ion count between 7-10 minutes with two peaks of $\mathbf{1}_{mod-2}$ (m/z = 944.36) at 9.25 and 9.36 minutes, of which mass fragmentation is presented in b) for the 9.20-9.25 minutes range, c) for the 9.36-9.45 minutes range and Table S5. Letters are used to label different fragments, with blue letters marking the fragments only present in one of the two spectra (>2% relative abundance). Based on these fragments, b) is assigned as the inner XGLK$_{mod-2}$FK modification and c) as the outer XGLKFK$_{mod-2}$.

Table S5: Calculated and found mass ions of the mass spectra in Figure S16, with corresponding fragment labeling. Entries in blue are only present in one of the two spectra (>2% relative abundance). Fragment names omit the charge and their endings are in grey to enhance readability.

Entry	Fragment	Chemical Formula	Calculated	Found in peak 1	Found in peak 2
a	NH_2-K-CO	$C_6H_{13}N_2O^+$	129.1022	129.1024	129.1024
b	unknown			231.1494	231.1495
c	NH_2-LK-CO	$C_{13}H_{26}N_3O_2^+$	242.1864	242.1865	242.1865
d	unknown			268.1004	268.1005
e	NH_2-FK-COOH	$C_{15}H_{24}N_3O_3^+$	294.1813	294.1815	294.1815
f	NH_2-K_{mod-2}-CO	$C_{14}H_{21}N_2O_4S^+$	313,1217	313.1219	313.1220
g	NH_2-K_{mod-2}-COOH	$C_{14}H_{23}N_2O_5S^+$	331.1322	n.d.	331.1327
h	NH_2-KFK-COOH	$C_{21}H_{36}N_5O_4^+$	422.2762	422.2765	422.2767
i	NH_2-LK_{mod-2}-CO	$C_{21}H_{34}N_3O_5S^+$	426.2058	426.2062	n.d.
j	XGLK-CO	$C_{21}H_{31}N_4O_4S_2^+$	467.1787	467.1788	467.1786
k	NH_2-FK_{mod-2}-CO	$C_{20}H_{32}N_3O_5S^+$	478.2009	478.1998	478.2012
l	NH_2-LKFK-COOH	$C_{27}H_{47}N_6O_5^+$	535.3602	535.3608	535.3610
m	XGLKF-CO minus CO	$C_{29}H_{40}N_5O_4S_2^+$	586.2517	n.d.	586.2529
n	unknown				596.2366
o	NH_2-$(KFK)_{mod-2}$-COOH	$C_{29}H_{44}N_5O_7S^+$	606.2956	606.2960	606.2960
p	XGLKF-CO	$C_{30}H_{40}N_5O_5S_2^+$	614.2471	614.2468	614.2471
q	XGLKF-CO plus H_2O	$C_{30}H_{42}N_5O_6S_2^+$	632.2572	n.d.	632.2577
r	$XGLK_{mod-2}$-CO	$C_{29}H_{39}N_4O_7S_3^+$	651.1981	651.1978	n.d.
s	NH_2-$(LKFK)_{mod-2}$-COOH	$C_{35}H_{55}N_6O_8S^+$	719.3797	n.d.	719.3804
t	XGLKFK-CO	$C_{36}H_{52}N_7O_6S_2^+$	742.3420	n.d.	742.3426
u	XGLKFK-COOH	$C_{36}H_{54}N_7O_7S_2^+$	760.3526	760.3524	760.3527
v	$XGLK_{mod-2}F$-CO minus CO	$C_{37}H_{48}N_5O_7S_3^+$	770.2710	770.2717	n.d.
w	$XGLK_{mod-2}F$-CO	$C_{38}H_{48}N_5O_8S_3^+$	798.2660	798.2665	n.d.
y	$XGLK_{mod-2}$ F-CO plus H_2O	$C_{38}H_{50}N_5O_9S_3^+$	816.2765	816.2772	n.d.
z	$(XGLKFK)_{mod-2}$-CO	$C_{44}H_{60}N_7O_9S_3^+$	926.3615	926.3622	926.3618

a

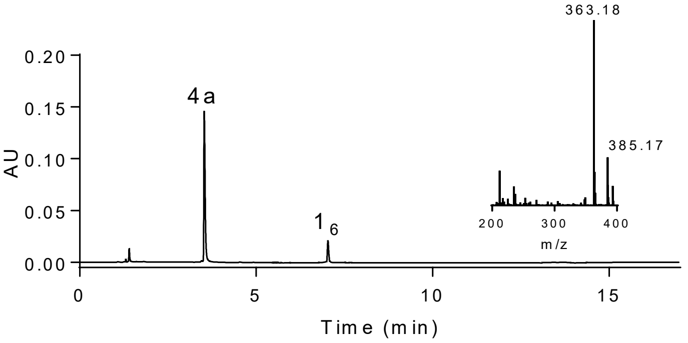

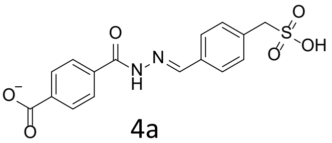

4a

	Calculated	Found
$[M+H]^+$	363.06	363.18
$[M+Na]^+$	385.05	385.17

b

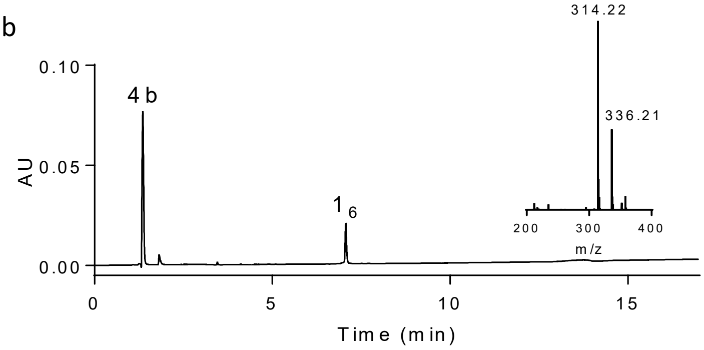

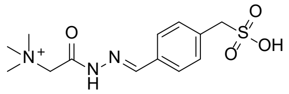

4b

	Calculated	Found
$[M+H]^+$	314.12	314.22
$[M+Na]^+$	336.10	336.21

c

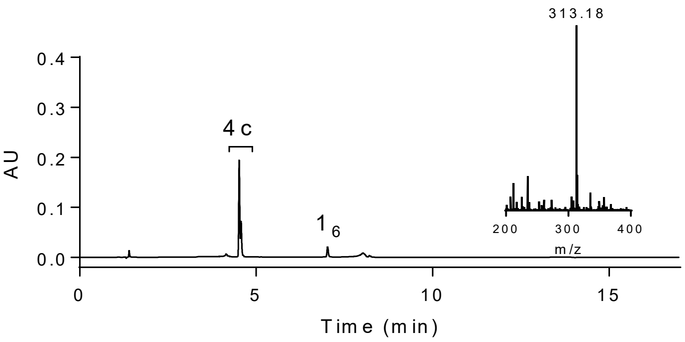

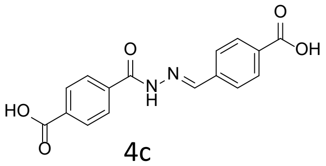

4c

	Calculated	Found
$[M+H]^+$	313.08	313.18

d

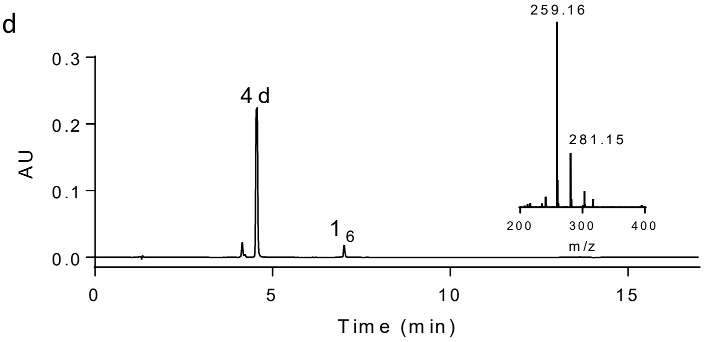

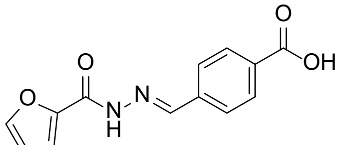

4d

	Calculated	Found
$[M+H]^+$	259.07	259.16
$[M+Na]^+$	281.05	281.15

e

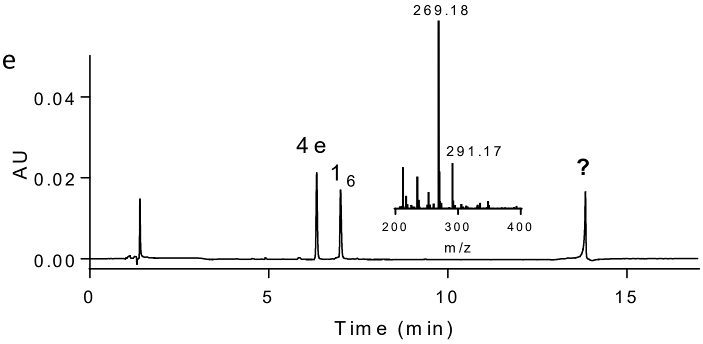

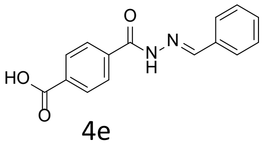

4e

	Calculated	Found
$[M+H]^+$	269.09	269.18
$[M+Na]^+$	291.07	291.17

f

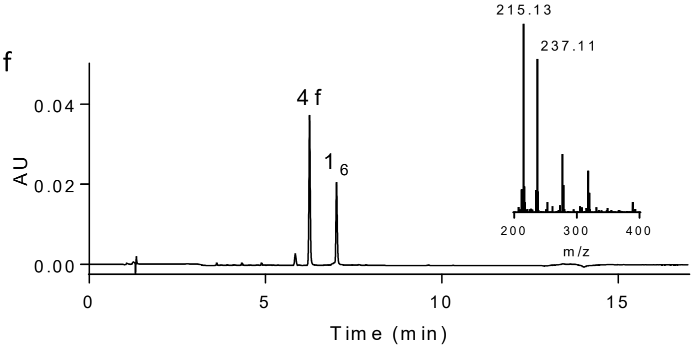

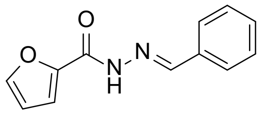

4f

	Calculated	Found
$[M+H]^+$	215.08	215.13
$[M+Na]^+$	237.06	237.11

g

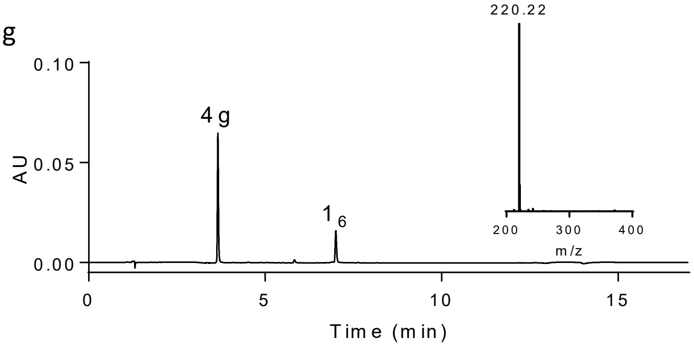

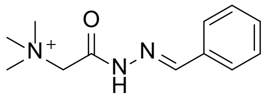

4g

	Calculated	Found
$[M]^+$	220.14	220.22

h

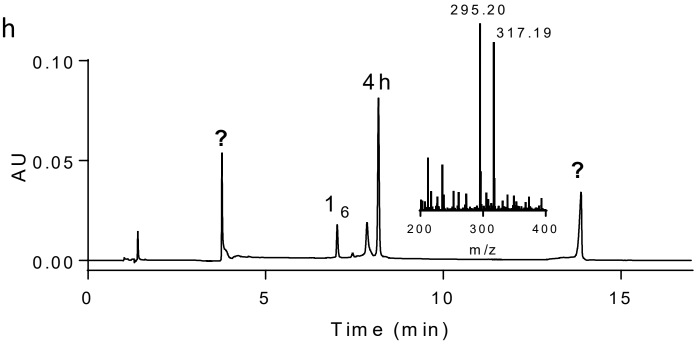

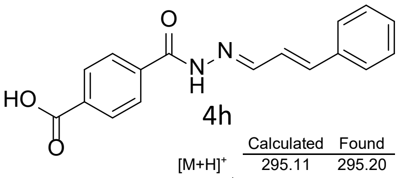

4h

	Calculated	Found
$[M+H]^+$	295.11	295.20
$[M+Na]^+$	317.09	317.19

i

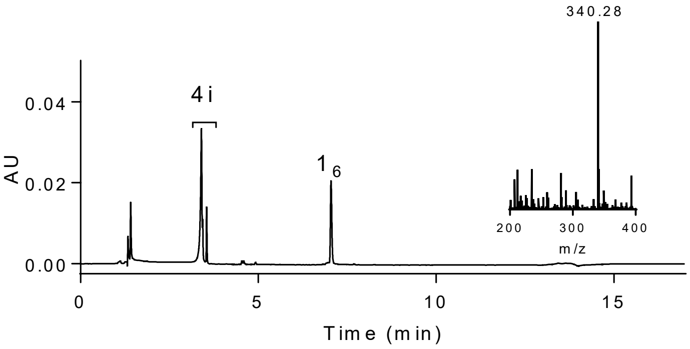

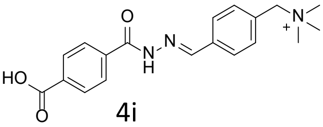

4i

	Calculated	Found
$[M]^+$	340.17	340.17

j

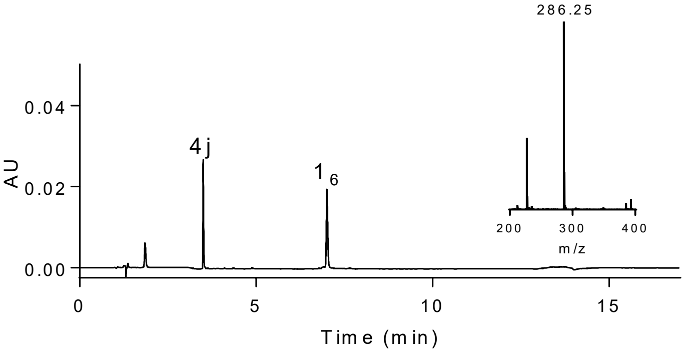

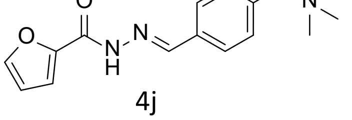

4j

	Calculated	Found
$[M]^+$	286.16	286.25

k

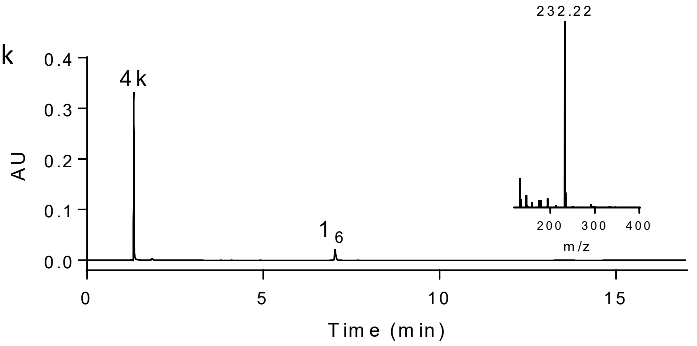

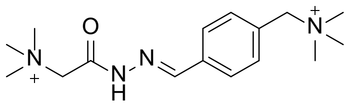

4k

	Calculated	Found
$[M-N(CH_3)_3^+]^{\cdot+}$	232.14	232.22
$[M]^{2+}$	146.11	146.17
$[M-H]^+$	291.42	291.31

l

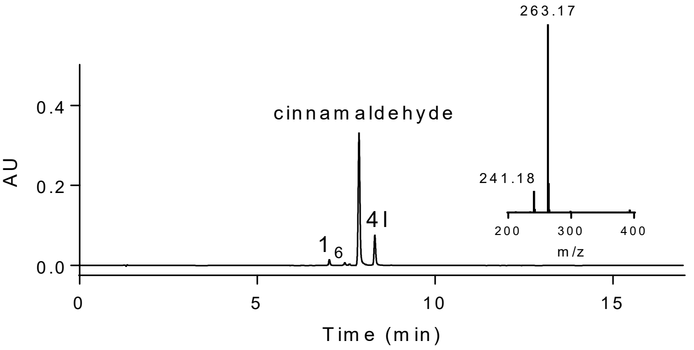

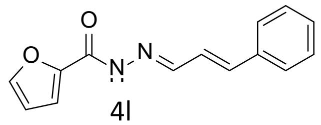

4l

	Calculated	Found
$[M+H]^+$	241.10	241.18
$[M+Na]^+$	263.08	263.17

m

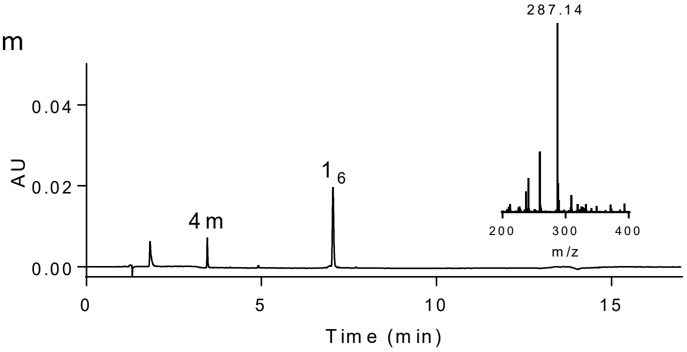

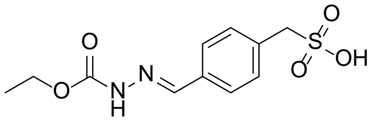

4m

	Calculated	Found
$[M+H]^+$	287.07	287.14

n

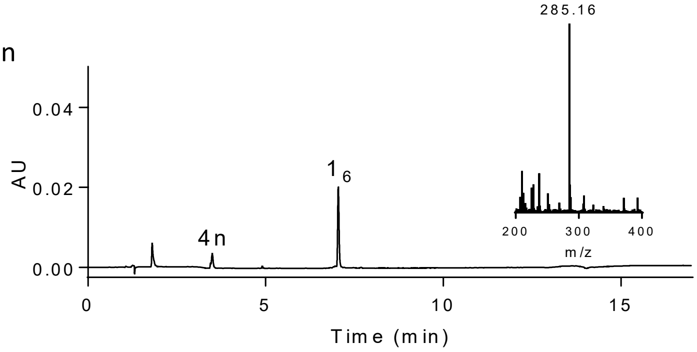

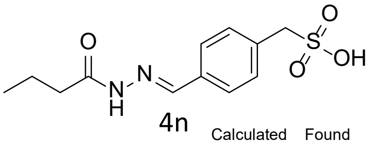

4n

	Calculated	Found
$[M+H]^+$	285.09	285.16

o

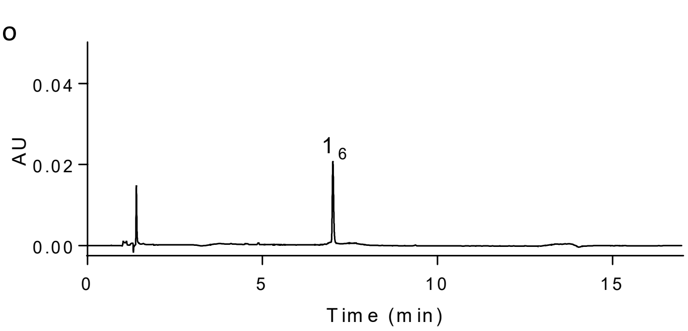

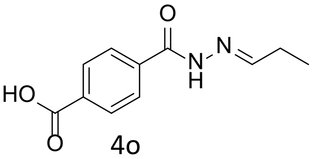

4o

p

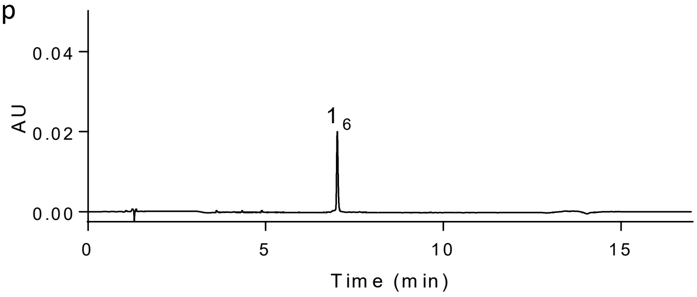

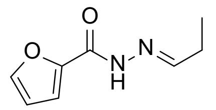

4p

Figure S17: a-p) UPLC chromatograms at 310 nm taken approximately 1 day after measurement of the ratio of catalyzed vs uncatalyzed initial reaction rate. Product formation was confirmed by UPLC-MS and insets show the partial mass spectrum of the ions corresponding to the respective products with indicated the calculated and found m/z values. For products **4m**-**4p** no clear increasing UV signal was obtained and only for **4m** and **4n** the product could be detected by UPLC & UPLC-MS. For **4c** and **4i**, a bracket is used to indicate the product as UPLC-MS (ESI+) did not resolve the peaks as well as the standalone UPLC instrument.

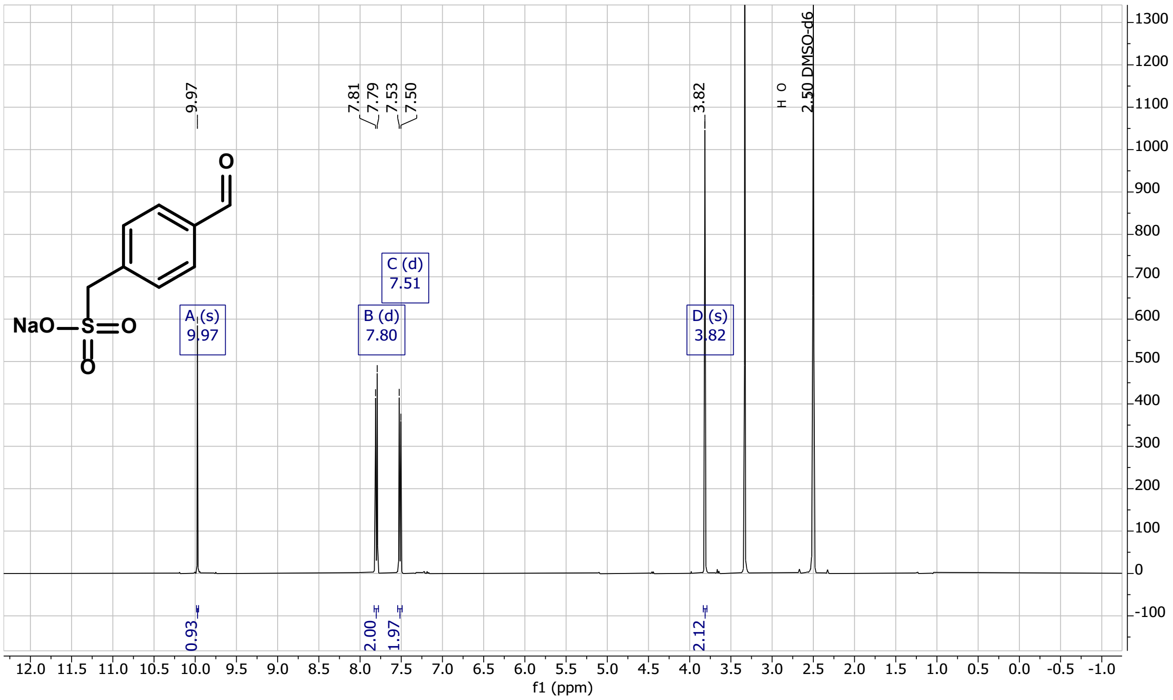

Figure S18: ^{1}H-NMR spectrum of **2** in DMSO-d$_6$.

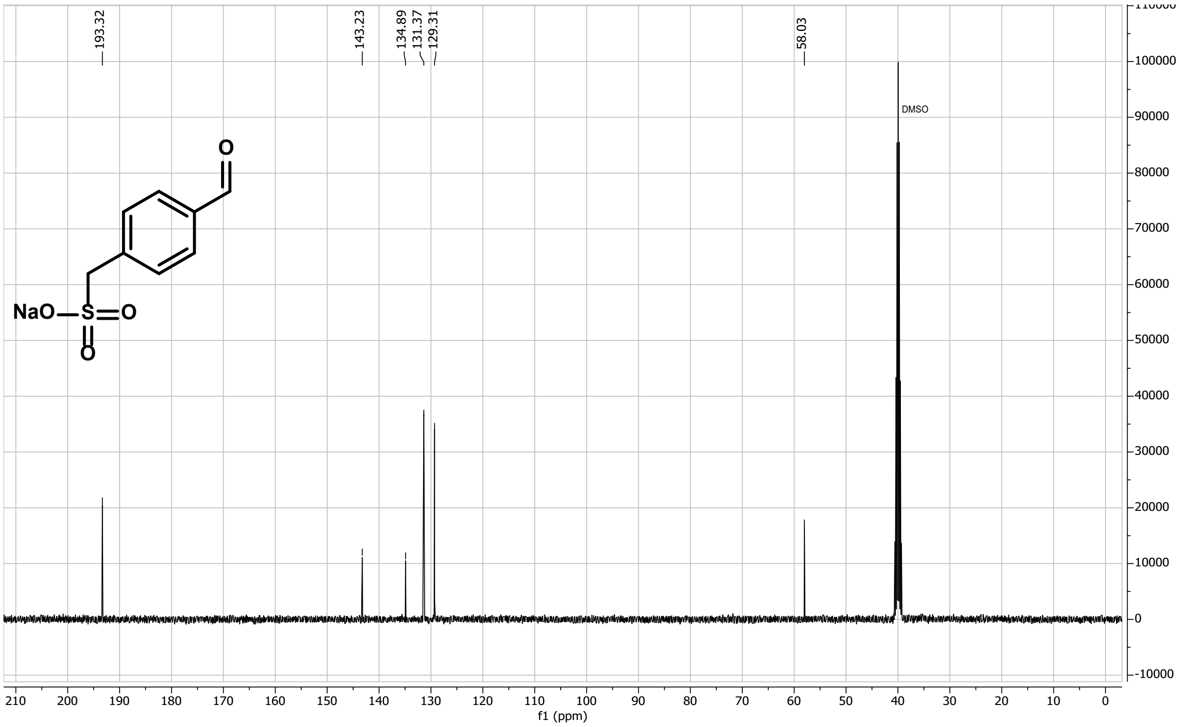

Figure S19: ^{13}C-NMR spectrum of **2** in DMSO-d$_6$.

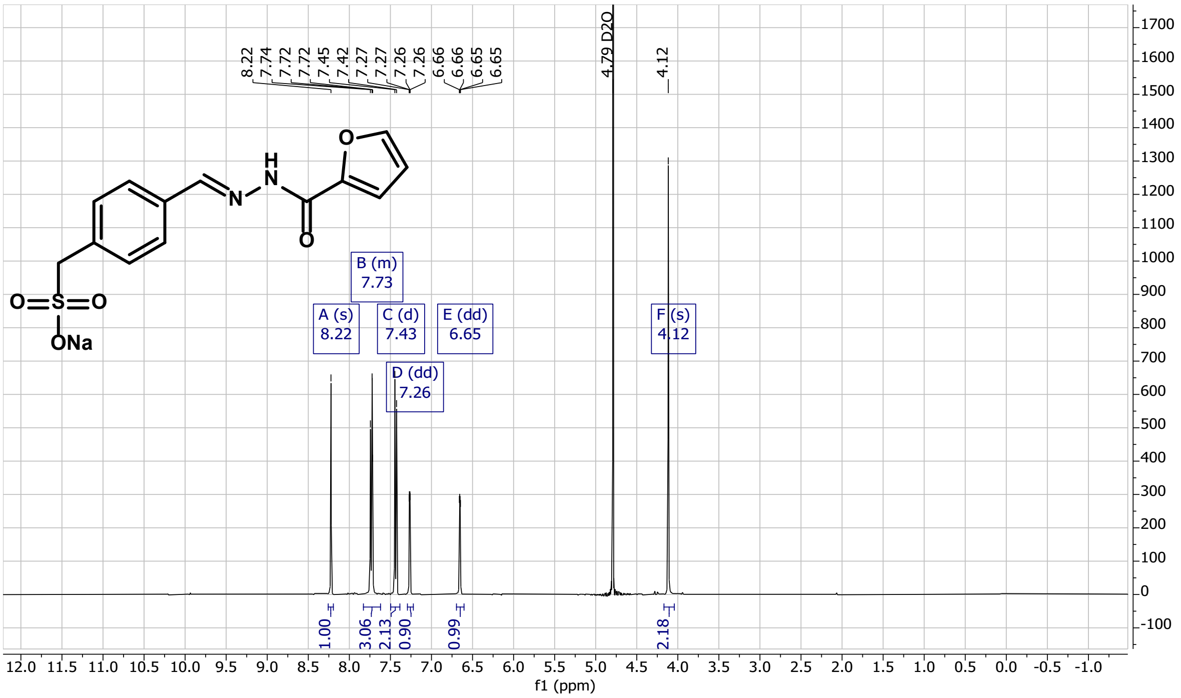

Figure S20: ^{1}H-NMR spectrum of **4** in D_2O.

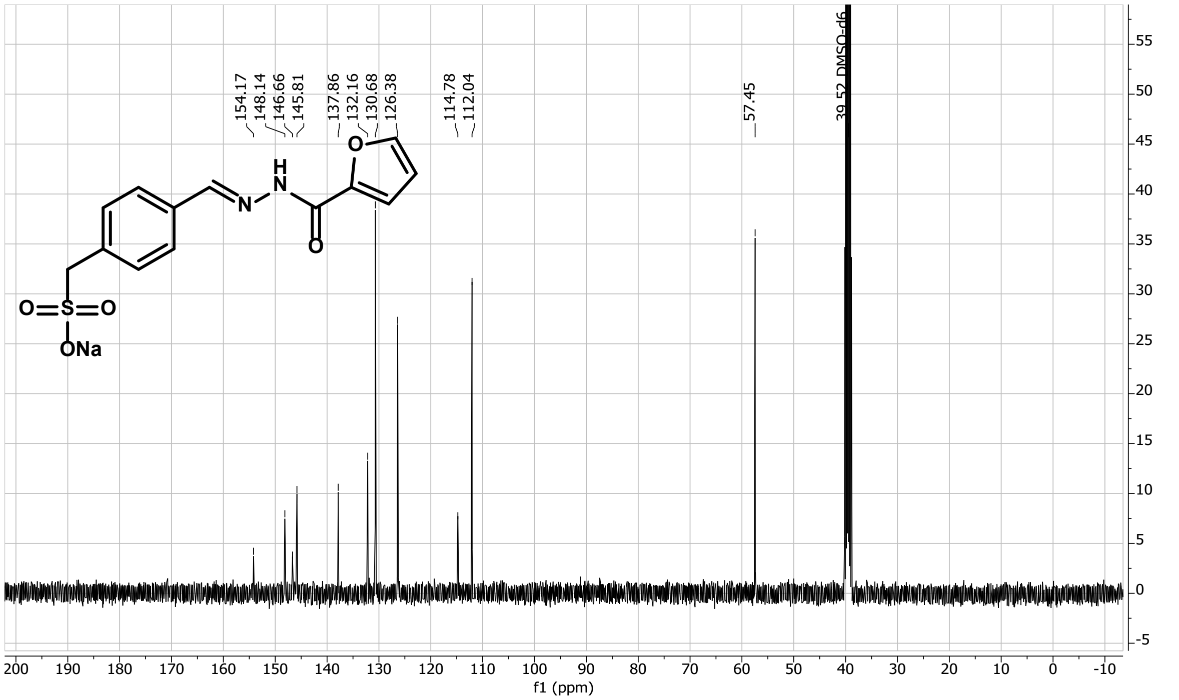

Figure S21: ^{13}C-NMR spectrum of **4** in DMSO-d$_6$.

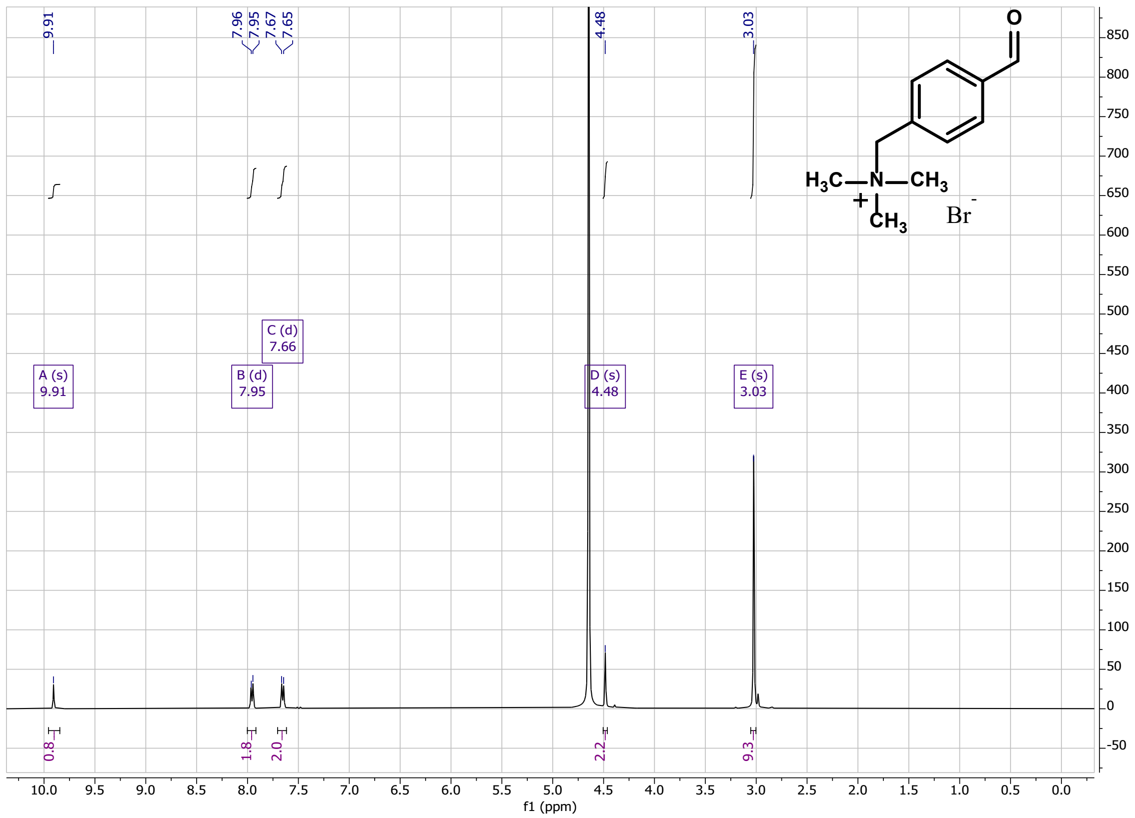

Figure S22: ^{1}H-NMR spectrum of (4-formylbenzyl)trimethylammonium bromide in D_2O.

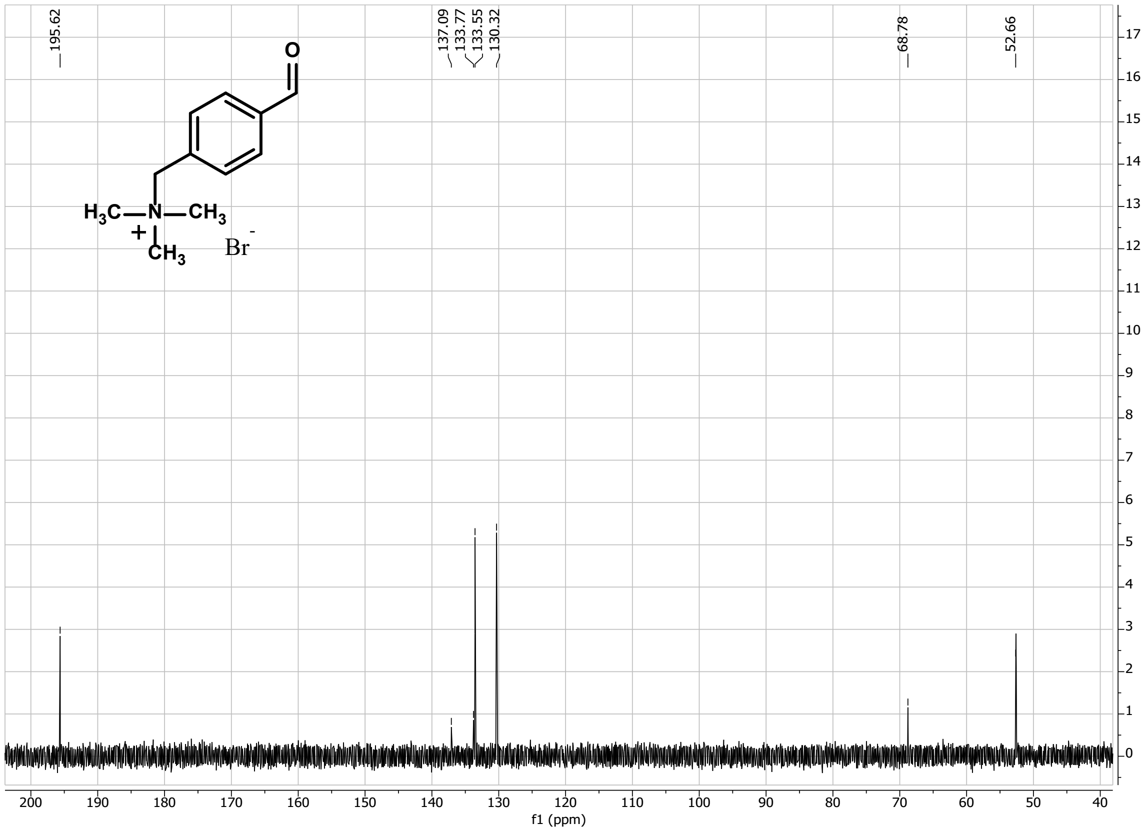

Figure S23: ^{13}C-NMR spectrum of (4-formylbenzyl)trimethylammonium bromide in D_2O.

17. Reference Tables

Table S6: Kinetic parameters of some designer enzymes for bimolecular reactions.

Entry	Name	Reaction	k_{uncat} (M^{-1} s^{-1})	k_{cat} (s^{-1})	$K_{m,S1}$ (M)	$K_{m,S2}$ (M)	k_{cat} / ($K_{m,S1}$ $K_{m,S2}$) ($M^{-2}s^{-1}$)	EM[a] (M)	1/K_{TS}[b] (M^{-1})
1[8]	**LmrR_pAF**	Acyl hydrazone	3.95 x10^{-4}	5.0 x10^{-4}	1.00 x10^{-4}	7.92 x10^{-3}	6.3 x 10^{2}	1.3	1.6 x10^{6}
2[9][c]	**LmrR_pAF_RMH**	Acyl hydrazone	3.95 x10^{-4}	4.53 x10^{-2}	4.8 x10^{-5}	4.64 x10^{-2}	2.05 x10^{4}	115	5.2 x10^{7}
3[9][c]	**LmrR_pAF_RMHL**	Acyl hydrazone	3.95 x10^{-4}	2.76 x10^{-2}	4.9 x10^{-5}	1.89 x10^{-2}	2.95 x10^{4}	69.8	7.5 x10^{7}
4[10]	**DA_20_00**	Diels Alder	6.8 x10^{-6}	2.8 x10^{-5}	3.5 x10^{-3}	1.5 x10^{-1}	5.4 x10^{-2}	2.3	7.9 x10^{3}
5[10][d]	**DA_20_10**	Diels Alder	6.8 x10^{-6}	5.9 x10^{-4}	1.3 x10^{-3}	7.3 x10^{-2}	6.2	87	9.2 x10^{5}
6[10][d]	**DA_42_04**	Diels Alder	6.8 x10^{-6}	8.3 x10^{-6}	5.0 x10^{-4}	1.6 x10^{-2}	1.03	0.70	1.5 x10^{5}
7[11]	**AB 39, A11**	Diels Alder	1.9	0.67	1.13 x10^{-3}	7.40 x10^{-4}	8.0 x10^{5}	0.35	4.2 x10^{5}
8[12][d]	**CE6**	Diels Alder	6.11 x10^{-6}	6.11 x10^{-4}	2.0 x10^{-4}	35 x10^{-3}	87	100	1.4 x10^{7}
9[13]	**AB 7D4**	Diels Alder	1.19 x10^{-5}	5.73 x10^{-5}	9.60 x10^{-4}	1.70 x10^{-3}	35	4.8	3.0 x10^{6}
10[13]	**AB 22C8**	Diels Alder	2.92 x10^{-6}	5.28 x10^{-5}	7.00 x10^{-4}	7.50 x10^{-3}	10	18	3.4 x10^{6}
11[14]	**AB 4D5**	Diels Alder	1.19 x10^{-5}	5.80 x10^{-5}	1.6 x10^{-3}	5.9 x10^{-3}	6.1	4.9	5.2 x10^{5}
12[14]	**AB 13G5**	Diels Alder	2.92 x10^{-6}	2.00 x10^{-5}	2.7 x10^{-3}	1.0 x10^{-2}	0.74	6.9	2.5 x10^{6}
13[15][e]	**BH32**	MBH[f]	-	3.6 x10^{-5}	8.0 x10^{-3}	1.8 x10^{-3}	2.5	-	-
14[15a][c,e]	**BH32.14**	MBH[f]	-	5.8 x10^{-3}	2.6 x10^{-3}	1.1 x10^{-3}	2.0 x10^{3}	-	-

[a] Effective molarity (EM = k_{cat}/k_{uncat}), [b] chemical proficiency ($1/K_{TS} = [k_{cat}/(K_{m,S1}\ K_{m,S2})]/k_{uncat}$), [c] engineered by directed evolution, [d] engineered by site directed mutagenesis, [e] apparent K_m values, [f] Morita-Baylis-Hillman reaction.

18. References

[1] a) S. Otto, R. L. E. Furlan, J. K. M. Sanders, *Science* **2002**, *297*, 590-593; b) J. M. A. Carnall, C. A. Waudby, A. M. Belenguer, M. C. A. Stuart, J. J.-P. Peyralans, S. Otto, *Science* **2010**, *327*, 1502-1506; c) L. Field, P. R. Engelhardt, *J. Org. Chem.* **1970**, *35*, 3647-3655.

[2] C. A. Schneider, W. S. Rasband, K. W. Eliceiri, *Nat. Methods* **2012**, *9*, 671-675.

[3] J. Ottelé, A. S. Hussain, C. Mayer, S. Otto, *Nat. Cat.* **2020**, *3*, 547-553.

[4] O. Markovitch, J. Ottelé, O. Veldman, S. Otto, *Commun. Chem.* **2020**, *3*, 180.

[5] S. Otto, J. B. F. N. Engberts, J. C. T. Kwak, *J. Am. Chem. Soc.* **1998**, *120*, 9517-9525.

[6] C. B. Minkenberg, L. Florusse, R. Eelkema, G. J. Koper, J. H. van Esch, *J. Am. Chem. Soc.* **2009**, *131*, 11274-11275.

[7] C. M. Davern, B. D. Lowe, A. Rosfi, E. A. Ison, C. Proulx, *Chem. Sci.* **2021**, *12*, 8401-8410.

[8] I. Drienovská, C. Mayer, C. Dulson, G. Roelfes, *Nat. Chem.* **2018**, *10*, 946-952.

[9] C. Mayer, C. Dulson, E. Reddem, A.-M. W. H. Thunnissen, G. Roelfes, *Angew. Chem. Int. Ed.* **2019**, *58*, 2083-2087.

[10] J. B. Siegel, A. Zanghellini, H. M. Lovick, G. Kiss, A. R. Lambert, J. L. St.Clair, J. L. Gallaher, D. Hilvert, M. H. Gelb, B. L. Stoddard, K. N. Houk, F. E. Michael, D. Baker, *Science* **2010**, *329*, 309-313.

[11] A. C. Braisted, P. G. Schultz, *J. Am. Chem. Soc.* **1990**, *112*, 7430-7431.

[12] C. B. Eiben, J. B. Siegel, J. B. Bale, S. Cooper, F. Khatib, B. W. Shen, F. Players, B. L. Stoddard, Z. Popovic, D. Baker, *Nat. Biotechnol.* **2012**, *30*, 190-192.

[13] V. E. Gouverneur, K. N. Houk, B. de Pascual-Teresa, B. Beno, K. D. Janda, R. A. Lerner, *Science* **1993**, *262*, 204-208.

[14] J. T. Yli-Kauhaluoma, J. A. Ashley, C.-H. Lo, L. Tucker, M. M. Wolfe, K. D. Janda, *J. Am. Chem. Soc.* **1995**, *117*, 7041-7047.

[15] a) R. Crawshaw, A. E. Crossley, L. Johannissen, A. J. Burke, S. Hay, C. Levy, D. Baker, S. L. Lovelock, A. P. Green, *Nat. Chem.* **2022**, *14*, 313-320; b) S. Bjelic, L. G. Nivón, N. Çelebi-Ölçüm, G. Kiss, C. F. Rosewall, H. M. Lovick, E. L. Ingalls, J. L. Gallaher, J. Seetharaman, S. Lew, G. T. Montelione, J. F. Hunt, F. E. Michael, K. N. Houk, D. Baker, *ACS Chem. Biol.* **2013**, *8*, 749-757.
